# Evaluating the Fluorescence Quenching of Troxerutin for Commercial UV Sunscreen Filters

**DOI:** 10.1021/acsphyschemau.4c00070

**Published:** 2024-10-25

**Authors:** Jack Dalton, Natércia d. N. Rodrigues, Daniel Berndt, Vasilios G. Stavros

**Affiliations:** † Department of Chemistry, University of Warwick, Gibbet Hill Road, Coventry CV4 7AL, U.K.; ‡ IBB-Institute for Bioengineering and Biosciences, Instituto Superior Técnico, Universidade de Lisboa, 1049-001 Lisboa, Portugal; § Symrise AG, 37603 Holzminden, Germany; ∥ School of Chemistry, University of Birmingham, Birmingham B15 2TT, U.K.

**Keywords:** energy transfer, FRET, dexter, fluorescence, quenching, ultrafast spectroscopy, photoprotection

## Abstract

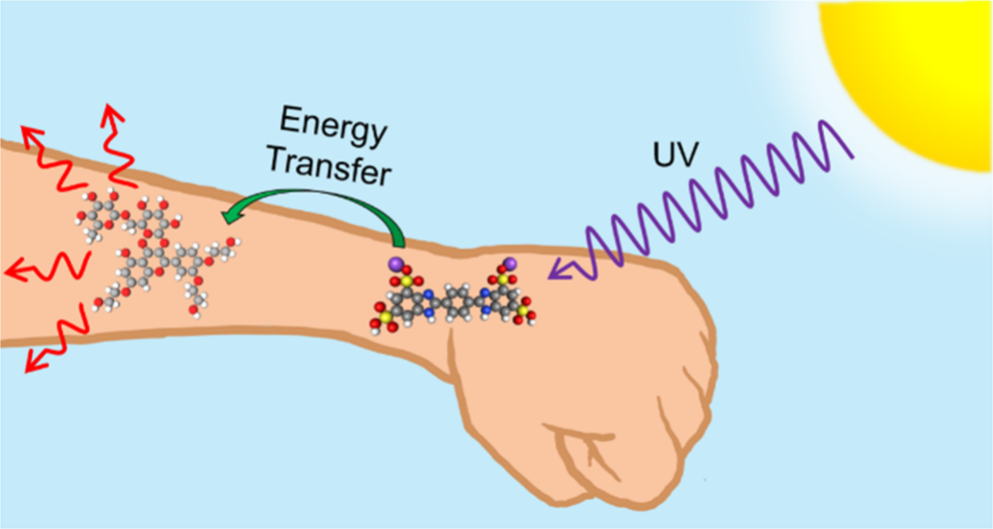

2-Phenylbenzimidazole-5-sulfonic acid (PBSA) and disodium phenyl dibenzimidazole tetrasulfonate (DPDT) are commercially available ultraviolet (UV) sunscreen filters that are known to undergo radiative relaxation following the absorption of UV light. The release of high-energy photons from this relaxation can be detrimental to human health; therefore, fluorescence quenchers need to be incorporated in commercial sunscreen formulations containing PBSA or DPDT. Troxerutin is a fluorescence quencher utilized for DPDT commercially. Here, its ability to quench the fluorescence of both PBSA and DPDT is evaluated using a dual-pronged approach by breaking down the multicomponent problem into its constituent parts. First, PBSA and DPDT’s femtosecond to nanosecond photodynamics are uncovered in solution and on the surface of a human skin mimic to ascertain a benchmark. Second, these results are compared to their photodynamics in the presence of troxerutin. A significant reduction in the fluorescence lifetime is observed for both PBSA and DPDT on a human skin mimic with the addition of troxerutin, which is attributed to a Dexter energy transfer (DET) or Förster resonance energy transfer (FRET) quenching mechanism. This finding demonstrates the hitherto unseen fluorescence quenching mechanism of troxerutin on a human skin mimic and its role in quenching the fluorescence of commercial UV sunscreen filters through a DET or FRET mechanism.

## Introduction

Ultraviolet (UV) radiation from the Sun, specifically UVA (315 to 400 nm) and UVB (280 to 315 nm), is both vital and detrimental to humans.^1^ A moderate exposure to UV radiation aids in maintaining healthy vitamin D levels in the body, which are known to help protect against several bone diseases, internal cancers, and multiple sclerosis.^2,3^ In contrast, overexposure to UV radiation can lead to sunburn, photoaging, and skin cancer.^4,5^ The body’s natural defense against this overexposure is melanogenesis, the production of melanin, commonly referred to as tanning.^6^ However, melanogenesis is a delayed response, and in most cases, photodamage has already occurred by the time any significant amount of extra melanin is produced. As such, an artificial defense often needs to be applied to the skin, commonly in the form of a commercial sunscreen formulation containing UV filters that absorb harmful UV solar radiation.^7^

Ideally, UV sunscreen filters should dissipate absorbed radiation quickly and efficiently, bypassing slow radiative relaxation (fluorescence or phosphorescence) and other pathways that may lead to photodegradation.^8,9^ The high-energy photons released from radiative decay can themselves be absorbed by the skin, resulting in the same detrimental effects above-mentioned, albeit to a lesser extent as the fluorescence occurs in all directions. Additionally, given the usually long lifetimes of radiative relaxation, there is an increased probability of the energy transferring to the surroundings and indirectly generating radicals and other reactive species. An inability to quickly dissipate excess energy may also lead to photodegradation of the UV filter, that is, the breakdown of the UV filter’s molecular structure, which ultimately leads to loss of the artificial defense and formation of potentially harmful photoproducts such as free radicals.^10^ Many commercially available UV filters fall short of the ideal photophysical behavior just described, particularly considering that, in addition to these photophysical requirements, UV filters must also pose no environmental risks.^11−15^ As such, it is imperative that new UV filters capable of the safe dissipation of absorbed energy or emission quenchers capable of efficient energy transfer are developed.

**Figure 1:**
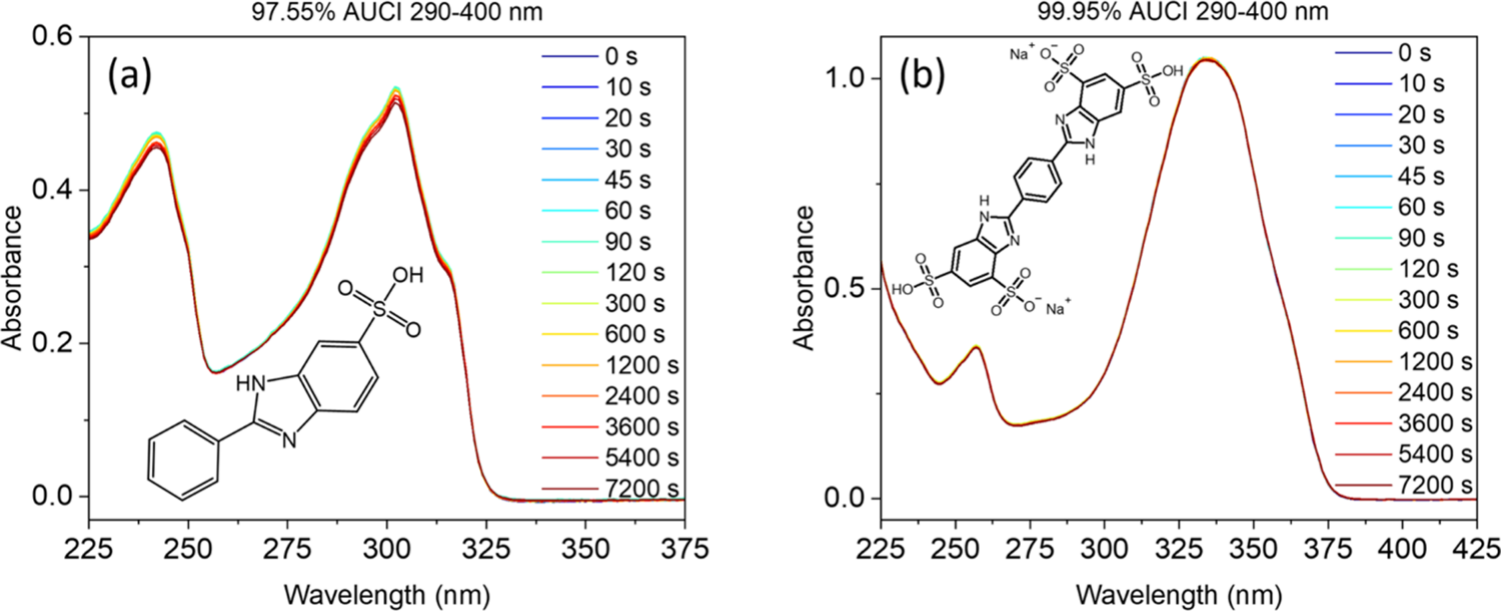
Absorption spectra of (a) 2-phenylbenzimidazole-5-sulfonic acid (PBSA) and (b) disodium phenyl dibenzimidazole tetrasulfonate (DPDT) at various times following solar simulator irradiation (see SI Figure S1 for simulated solar spectrum). The path length is 1 cm, and both solutions are at 20  μM in water and then neutralized with NaOH. The structures of PBSA and DPDT are shown in the inset of (a, b), respectively. The area under the curve index (AUCI) between 290 and 400 nm is shown as a percentage at the top of each graph.

2-Phenylbenzimidazole-5-sulfonic acid (PBSA, see Figure 1a for structure), commonly known as ensulizole, is a commercially available UVB filter in use worldwide.^7^ Neutralizing the acid into its corresponding salt with a base such as NaOH turns PBSA from a UV filter with poor water solubility into one of the very few water-soluble UV filters on the market. At first glance, PBSA proves promising as an ideal UV filter with strong (∼25,000 M^–1^ cm^–1^, depending on counterion and solvent) and broad UVB absorption, as also shown in Figure 1a.^16−18^ However, this molecule’s primary mechanism for dissipation of absorbed UV energy is via slow radiative relaxation, with peak emission at 343 nm and a known fluoresce quantum yield of 0.63 (in phosphate buffer, pH 7.4).^19^ Consequently, this has stimulated study into methods of quenching this fluorescence, particularly using quantum dots.^20−25^

**Figure 2:**
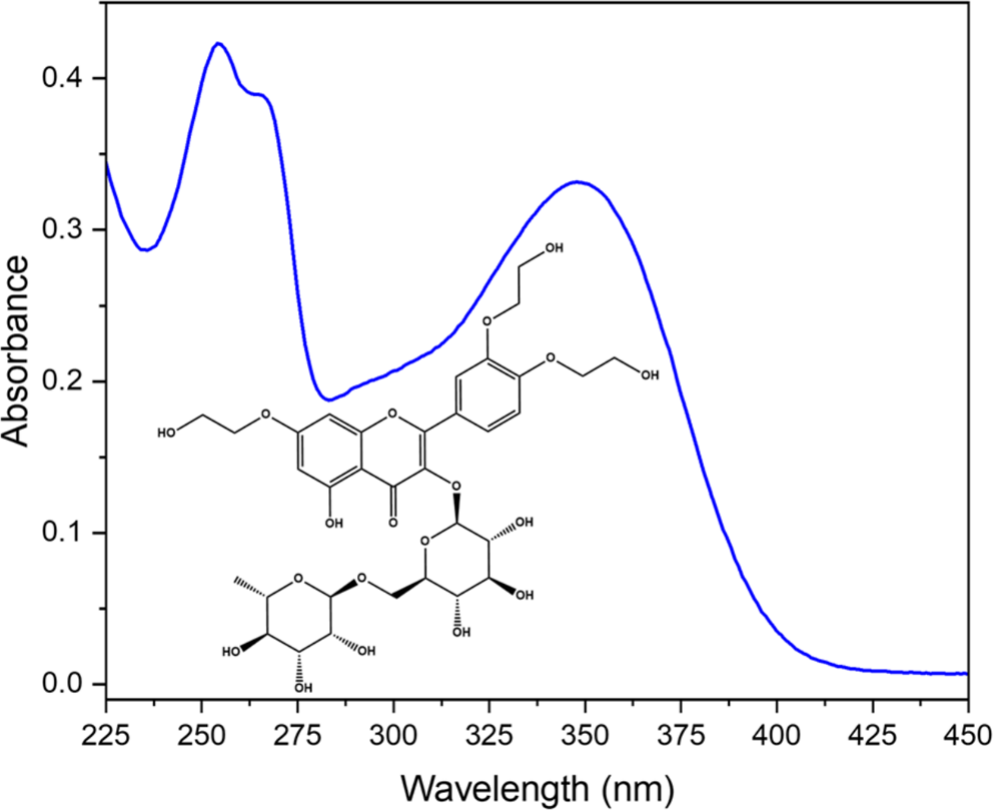
Absorption spectrum of 20 μM troxerutin in water. The molecular structure of troxerutin is shown in the inset. The path length is 1 cm.

A close structural analogue of PBSA, disodium phenyl dibenzimidazole tetrasulfonate (DPDT, see Figure 1b for structure), is another commercially available UV filter that is approved for use in the EU and Australia.^7^ The salt of DPDT is likewise highly water-soluble, and its extended structure red-shifts its peak absorption relative to PBSA into the UVA region, in addition to approximately doubling the peak absorption strength (∼50,000 M^–1^ cm^–1^, depending on counterion and solvent), all while maintaining broad UV coverage.^26,27^ Similarly to PBSA, DPDT is known to emit light following UV absorption, although very few studies have investigated possible quenching approaches for this species.^26,28^ For commercial use, the developers of DPDT, Symrise (Holzminden, Germany), have patented the use of troxerutin for the emission quenching of DPDT (see Figure 2 for molecular structure).^29^ Although quenching has been observed and patented, the mechanism of quenching for troxerutin with DPDT has not been reported in the literature. For various fluorescent biological samples, troxerutin has demonstrated quenching through a static quenching mechanism; however, this is under very different conditions from that experienced by UV sunscreen filters.^30−34^

The work presented here seeks to investigate the use of troxerutin as an emission quencher on two commercial UV sunscreen filters, PBSA and DPDT. In order to understand the mechanism of quenching, one needs to first break down the multicomponent problem into individual components. As such, the femtosecond to nanosecond photodynamics of PBSA and DPDT are initially investigated in solution and on a human skin mimic. Following this, their photodynamics are compared to that seen in the same conditions with the addition of troxerutin; a multipronged experiment approach comprising ultrafast transient electronic absorption spectroscopy (TEAS), time-resolved emission spectroscopy, and steady-state absorption and emission spectroscopy was used. These techniques reveal static and time-resolved spectra, relaxation lifetimes, and emission quantum yields to ultimately assemble a picture of the events following the absorption of UV light. From these results, we demonstrate that both PBSA and DPDT relax primarily via an unfavorable fluorescence relaxation pathway, to which troxerutin can quench this emission on a skin surface via a Dexter energy transfer or Förster resonance energy transfer mechanism. The results of the present work not only highlight the potential hazards that current commercial UV filters pose but, more importantly, reveal the fluorescence quenching mechanism troxerutin follows on the surface of the skin.

## Results and Discussion

### In Solution

The UV–visible absorption spectra before and after various durations of simulated solar irradiation for PBSA and DPDT in water (20 μM; all water samples were neutralized with NaOH) are shown in Figure 1a,b, respectively. PBSA displays a UVB absorption maximum (λ_max_) of 302 nm and a molar extinction coefficient (ε) of ∼26,500 M^–1^ cm^–1^, which strongly correlates to the S_1_ (lowest-unoccupied molecular orbital (LUMO) ← highest occupied molecular orbital (HOMO)) excited state previously calculated by Shen.^35^ Shen’s time-dependent density-functional theory (TD-DFT) calculations reveal a large oscillator strength (0.89) for the S_1_ state and a small oscillator strength for nearby singlet states (the closest of these being >20 nm above the S_1_), which suggests that the prominent shoulder features in the absorption spectrum of PBSA (Figure 1a) are a result of a vibronic progression, not other electronic states. Compared to PBSA, the λ_max_ of DPDT red shifts to a UVA absorption of 334 nm and ε doubles to ∼52,500 M^–1^ cm^–1^. These changes may suggest that either DPDT behaves as 2 distinct chromophores (2 PBSA-like molecules in one), with the red shift being caused by the addition of the sulfonate group, or that the conjugation extends across the whole molecule, reducing the energy of the excited state, with ε being coincidentally increased 2-fold. Following irradiation of PBSA and DPDT with a solar simulator for 2 h (solar simulator spectrum shown in Supporting Information, SI, Figure S1), both molecules perform remarkably well, with area under the curve indexes (AUCI) between 290–400 nm of 97.55 and 99.95%, respectively (where AUCI = 100% × final AUC/initial AUC). An AUCI of >80% has previously been cited as a criterion for a photostable molecule, i.e., a molecule whose molecular integrity is maintained despite irradiation.^36,37^ The high photostability of these UV filters ensures that adequate UV protection is preserved for extended periods of time in direct sunlight while also negating the direct formation of potentially harmful photoproducts resulting from molecular degradation of the UV filter.

**Figure 3:**
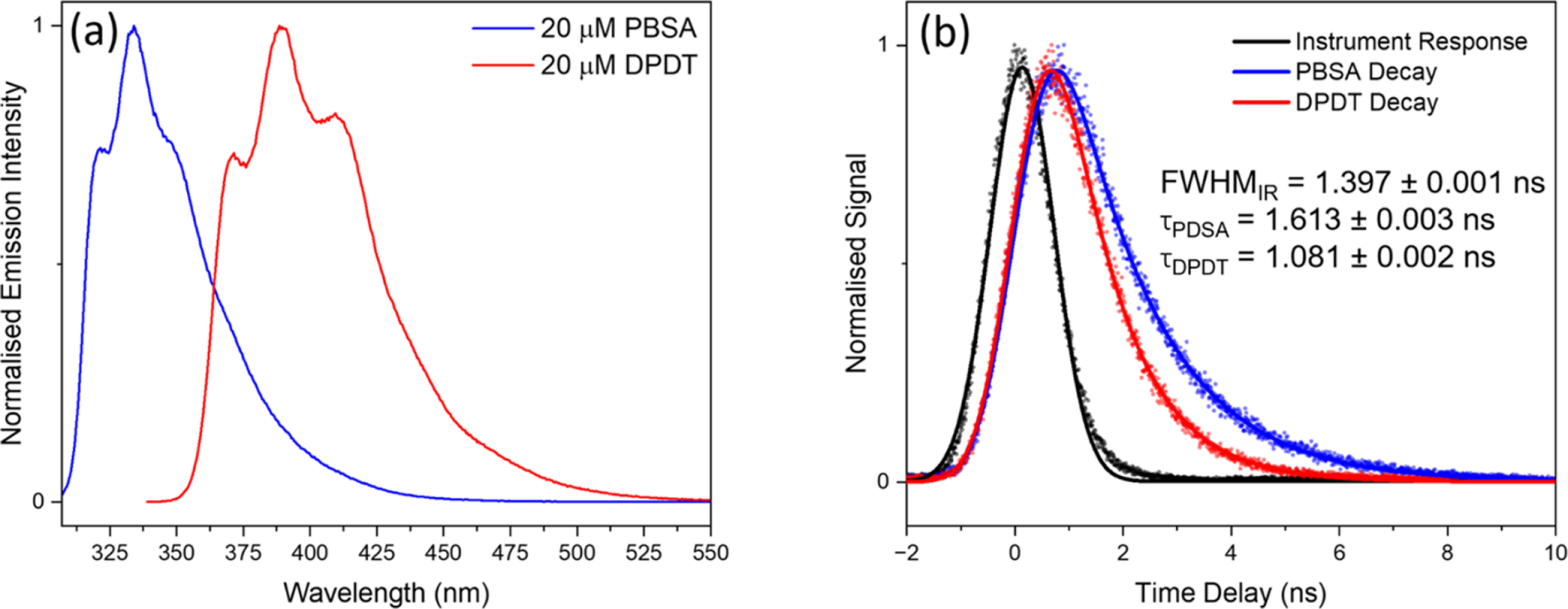
Emission studies of PBSA and DPDT in water (following neutralization with NaOH) at 20 μM in a 1 cm quartz cuvette. (a) The normalized emission spectrum of PBSA (blue) and DPDT (red) following excitation at their absorption maximum (λ_max_), 302 and 334 nm, respectively. (b) Emission lifetime measurements of PBSA (blue dots) and DPDT (red dots) following excitation at 318 nm and detection at their respective emission maximum. Black dots in (b) show the instrument response (IR) of the experiment obtained by measuring the 318 nm pump scatter signal in water. A Gaussian fit of the IR (black line) has a full width at half-maximum (fwhm_IR_) of 1.397 ns. The decay in the emission of PBSA and DPDT was fit with a monoexponential decay convoluted with a Gaussian and returned lifetimes of 1.613 ± 0.003 and 1.081 ± 0.002 ns, respectively. The errors shown relate to one standard error between the fit and the raw data.

As discussed above, following the absorption of UV light, these molecules are known to dissipate excess energy via the emission of radiation. Figure 3a displays the emission spectra of PBSA and DPDT at 20 μM following excitation at their λ_max_. The peak emission wavelengths of PBSA and DPDT are 334 and 389 nm, respectively, and both molecules exhibit characteristic vibronic progressions in their emission profiles. Monitoring the peak emission of these molecules as a function of time produces emission lifetimes of 1.613 ± 0.003 and 1.081 ± 0.002 ns for PBSA and DPDT, respectively (see Figure 3b), where the quoted errors relate to one standard error between the fit and the raw data. The emission lifetime of PBSA agrees with that seen previously by Mubeen et al., while, to the best of our knowledge, this is the first reported emission lifetime for DPDT.^24^ Additionally, no change in the lifetimes for both PBSA and DPDT was found when analogous measurements were carried out under a nitrogen atmosphere, thus confirming fluorescence and not phosphorescence as the primary radiative pathway (see Figure S2 in the SI).

The fluorescence quantum yields of both PBSA and DPDT in water at 20 μM were also determined, as presented in the SI, Figure S3. For PBSA, the fluorescence quantum yield is 72%. This is comparable to the 63% that was found for PBSA in a phosphate buffer, confirming that the major relaxation mechanism of PBSA is fluorescence.^19^ However, this indicates a possible dependence of fluorescence quantum yield on the salt’s cation and, as such, implies that the surrounding environment could have a significant impact on the relaxation mechanism of PBSA. For DPDT, a fluorescence quantum yield of 94% was determined. As with PBSA, this confirms that DPDT’s major relaxation mechanism is via fluorescence. These high levels of radiative decay in the absence of a quencher raise concerns about the use of either UV filter in commercial sunscreen formulations.

**Figure 4:**
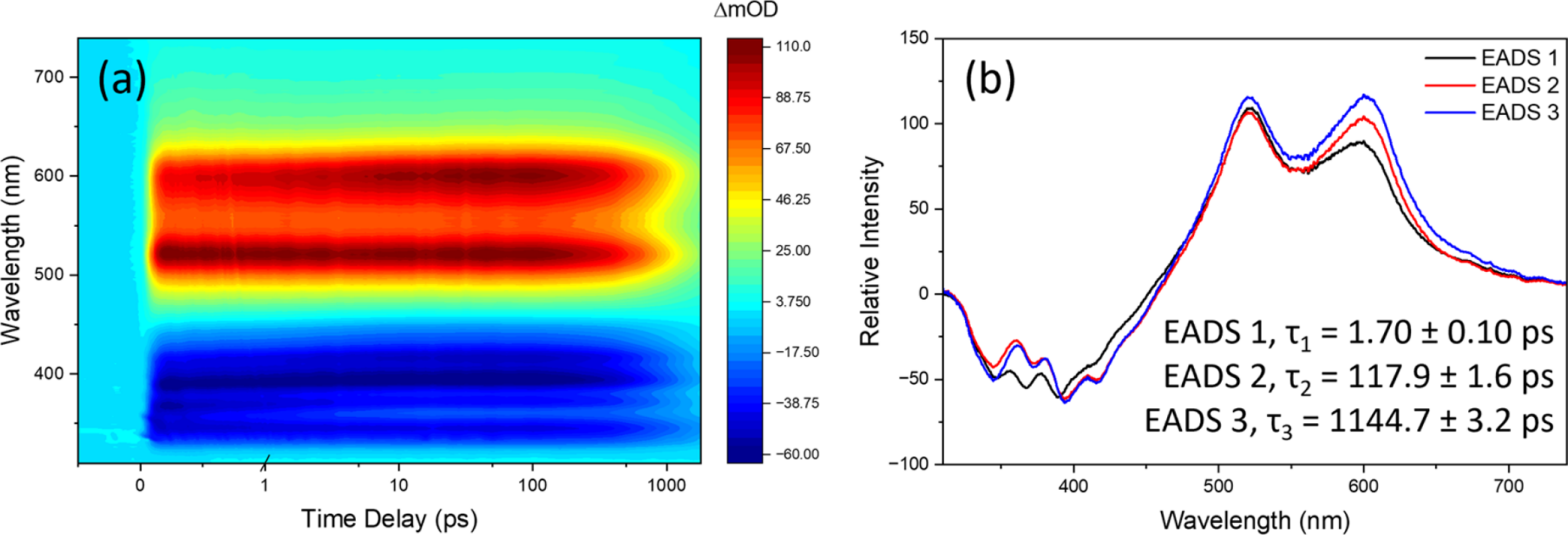
TEA spectra presented as a false color heatmap for (a) DPDT at 500 μM in water (neutralized with NaOH), following excitation at λ_max_, 334 nm. The time delay is linear up to 1 ps and logarithmic from 1 to 1800 ps. The evolution-associated difference spectra (EADS) obtained from the global sequential fitting model in the Glotaran software package (see the Experimental Section for further details) are shown in (b), along with the corresponding lifetimes.

Complementary TEAS measurements were carried out to investigate the photodynamics of these UV filters, aiming specifically to explain their lack of fast, nonradiative decay pathways, which are preferable in sunscreen filters. The transient electronic absorption (TEA) spectra obtained for DPDT in water at 500 μM following excitation at 334 nm are presented in Figure 4a. Three main features can be identified: the first is a negative feature at ∼340 nm that decays alongside the other features presented in the heatmap. This spectral feature closely resembles that of the absorption spectrum (Figure 1b), and it is therefore assigned to ground-state bleach (GSB). The second feature is also negative and has three distinct peaks, with the central peak at the highest intensity at ∼390 nm. By comparison with the emission spectrum in Figure 3a, this feature is attributed to the stimulated emission (SE). As DPDT relaxes, the SE feature displays a minor red shift of ∼5 nm. The final third feature is positive and consists of two peaks at ∼520 and ∼600 nm. This feature is assigned to excited-state absorption (ESA) to either two close-lying electronic states or to two vibronic bands. Interestingly, the ∼520 nm ESA peak stays relatively consistent in its spectral position and intensity, while the ∼600 nm ESA peak has a small red shift (∼3 nm) and grows in intensity to a maximum at ∼100 ps. The growth of the ∼600 nm ESA suggests structural changes that result in an increase in the transition strength between the populated excited state and the higher-lying state responsible for this feature.

To extract kinetic information from the TEA spectra, a global sequential decay fitting model was employed through the Glotaran software package;^38^ the lifetimes extracted from this fitting are summarized in Table 1. For DPDT in water, this returns three lifetimes: τ_1_ = 1.70 ± 0.10 ps, τ_2_ = 117.9 ± 1.6 ps, and τ_3_ = 1144.7 ± 3.2 ps (see SI Figure S4(e) for fitting residuals). The corresponding evolution-associated difference spectra (EADS), where spectral changes are more apparent, are presented in Figure 4b. Each EADS is associated with each corresponding lifetime; EADS_1_ decays within τ_1_ with a small but evident spectral red shift in the SE feature. This is concomitant with an increase in the intensity of the ∼600 nm ESA feature. These spectral changes suggest that, within τ_1_, the energy gap between the initially populated excited state and the electronic ground state is reduced, and the Franck–Condon overlap for the transition accounting for the ∼600 nm ESA feature increases. Considering this, τ_1_ is assigned to the initial vibrational and geometry relaxations in the molecule before arriving at a local minimum. EADS_2_, the corresponding EADS for the second lifetime, τ_2_, displays further spectral shifts, albeit minor, in both the SE and the ESA features, with additional intensity changes in the ESA features. This suggests further structural changes within the system, and as such, from the local minimum, τ_2_ is assigned to subsequent additional vibrational and geometry relaxation, possibly involving a non-negligible energy barrier along the reaction coordinate. While we recognize this to be uncharacteristically slow for vibrational relaxation, it is possible that we are observing dynamics related to the considerably large solvation shell around DPDT, which might be particularly strongly bound due to the charged character of the solute.^39,40^ The final lifetime, τ_3_, of 1144.7 ± 3.2 ps coincides with the emission lifetime of 1.081 ± 0.002 ns seen in Figure 3b, and the emission spectrum matches the SE feature seen in EADS_3_. As such, τ_3_ is assigned to fluorescence from a potential energy minimum on the initially populated singlet state to the electronic ground state.

**Table 1:** Tabulated Lifetimes of DPDT and PBSA in Water at 500 μM and on Skin in the Absence and Presence of Troxerutin

	DPDT			PBSA	
	τ_1_/ps	τ_2_/ps	τ_3_/ps	τ_1_/ps	τ_2_/ps
water	1.70 ± 0.10	117.9 ± 1.6	1144.7 ± 3.2	56.6 ± 0.6	1676.8 ± 6.2
skin	1.09 ± 0.10	55.4 ± 1.6	616.1 ± 6.5	44.2 ± 1.2	949.3 ± 7.9
water + troxerutin	1.25 ± 0.10	115.6 ± 2.0	1145.6 ± 4.1	48.8 ± 0.7	1608.1 ± 6.0
skin + troxerutin	1.61 ± 0.10	47.7 ± 1.0	389.0 ± 4.0	16.2 ± 0.4	536.0 ± 4.2

The TEAS measurements of PBSA in water at 500 μM following excitation at 302 nm display an overall relaxation mechanism similar to DPDT (see SI Figure S5 for heatmaps, EADS, and fitting residuals); hence, the results are only briefly discussed here. Two spectral features are seen in the TEA spectra of PBSA: the first is negative and peaks at ∼340 nm, and the second is positive and peaks at ∼430 nm. Following the same reasoning as that with DPDT, these features are assigned to SE and ESA, respectively. Similarly to DPDT, there are very minor spectral shifts along with changes in ESA intensity over time. The spectra are fitted with two lifetimes (see Table 1), the first of which is assigned to vibrational and geometry relaxation to a potential energy minimum, and the second is assigned to subsequent fluorescence to the electronic ground state. The former is supported by the spectral changes, while the latter is supported by the coinciding emission lifetime observed in Figure 3b.

In summary, we confirm that both molecules dissipate the energy absorbed upon UV radiation primarily through radiative decay pathways, namely, fluorescence. The photodynamical behavior observed in our experiments suggests that the potential energy landscape for these molecules offers no readily available nonradiative decay pathways, possibly due to relative structural rigidity (the inability to fold or twist into significantly different conformers) impeding broader molecular motion and thus precluding internal conversion. As a result, the excited-state population becomes trapped in the excited state, from which deactivation takes place almost exclusively via photon release. Nevertheless, we have also not found evidence for any dissociative or other photochemical pathways, meaning that the structural integrity of these UV filters is maintained upon irradiation, an important characteristic for filters to be used in commercial formulations.

### On Skin

**Figure 5:**
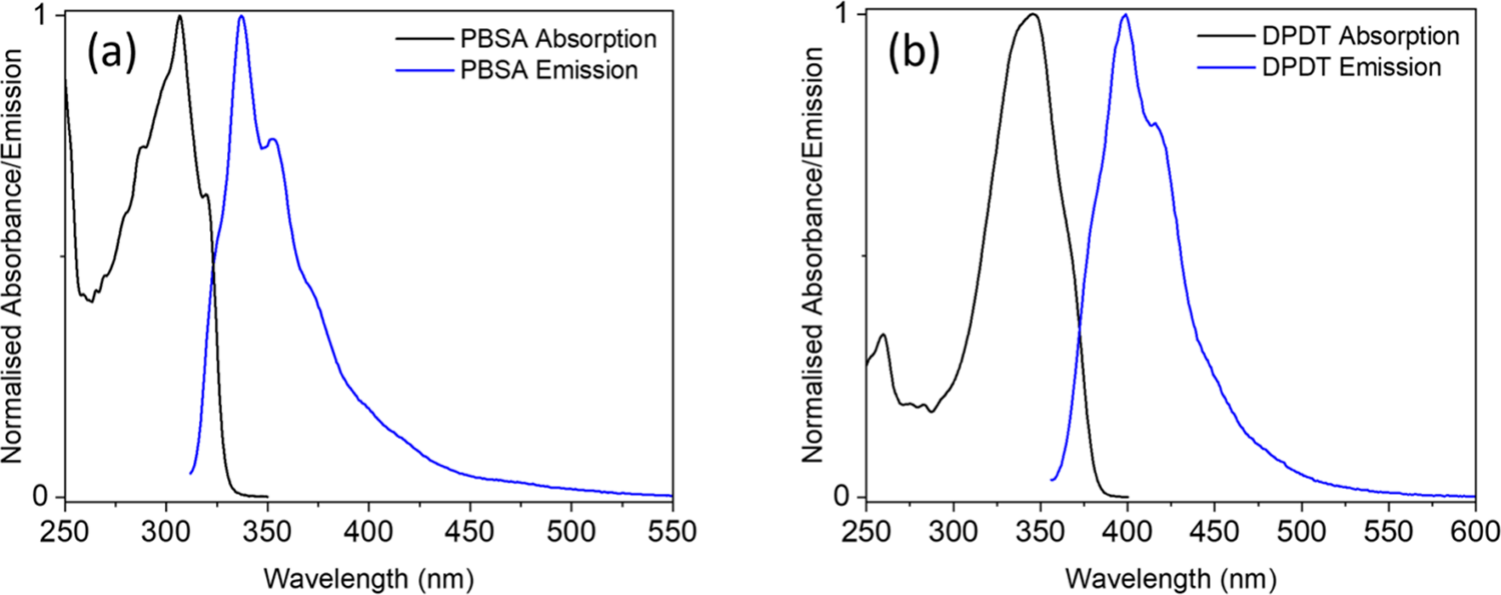
Absorption (black) and emission (blue) spectra of (a) PBSA and (b) DPDT on a synthetic skin mimic, VITRO–CORNEUM. The emission spectra are produced following excitation at each molecule’s respective λ_max_.

Absorption, emission, and TEAS measurements were taken of PBSA and DPDT on a synthetic skin mimic, VITRO–CORNEUM (see the Experimental Section for sample preparation details), to investigate the effects of a more realistic environment on the observed photodynamics. Figure 5a,b shows the absorption and emission spectra of PBSA and DPDT, respectively, on the synthetic skin mimic. Compared to the spectra obtained in solution, a red shift in both absorbance and emission is noticeable, with λ_max_ values for PBSA and DPDT now located at 307 and 346 nm, respectively (cf. 302 and 334 nm in solution), and a peak emission of 337 and 399 nm, respectively (cf. 334 and 389 nm in solution). This red shift in absorbance is desirable for sunscreen molecules as it results in the molecules absorbing more UVA from the Sun, a known challenge for the industry.^41^ This spectral shift hints at a change in the energy difference between the ground and excited states, which might have an impact on the mechanisms through which these molecules dissipate excess energy. Furthermore, the spectra show a near mirror-image relationship between the absorption and emission on the skin mimic. This indicates only minor structural changes on the skin mimic, which could arise due to the steric effects imposed by the skin.

**Figure 6:**
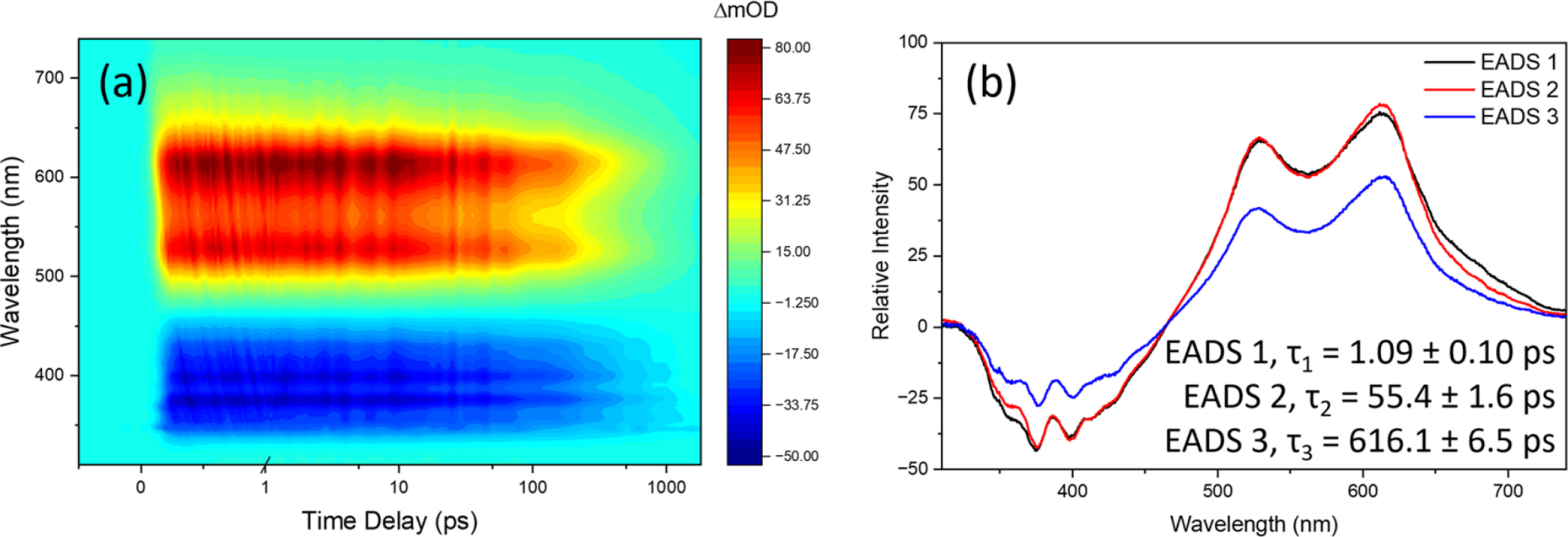
TEA spectra presented as a false color heatmap for (a) DPDT on human skin mimic, following excitation at λ_max_. The time delay is linear up to 1 ps and logarithmic from 1 to 1800 ps. The EADS obtained from the global sequential fitting model in the Glotaran software package (see the Experimental Section for further details) is shown in (b), along with the corresponding lifetimes.

Analogous to solution-phase TEAS measurements, the TEA spectra of both PBSA and DPDT display similar findings, and the following sections will focus on DPDT. Figure 6a shows the TEA spectra of DPDT on the skin following excitation at 346 nm. Broadly, the spectra exhibit features similar to that seen in solution, and, as such, they are assigned likewise. Relative to water, the two ESA features are both red-shifted by ∼10 nm to ∼530 and ∼610 nm, and the SE and GSB features are red-shifted by ∼10 nm in accordance with the emission and absorption spectrum (Figure 5b). This suggests that the interaction with skin has an impact on the energy landscape of the electronic excited states, also evidenced by the fairly constant intensity of the ESA and SE features, which indicates an unvarying Franck–Condon overlap between states responsible for these features, an observation in contrast to what we observe in solution. This, in addition to the features displaying fewer spectral shifts during relaxation than in solution, implies that the molecule undergoes less vibrational and geometrical relaxation, as would be expected from the presence of a rigid surface structure likely to restrict molecular motion. This finding is consistent with the near mirror-image relationship between the absorption and emission spectra on the skin mimic (Figure 5).

The lifetimes and corresponding EADS for DPDT on the skin from the global sequential fitting model are shown in Table 1 and Figure 6b, respectively (see SI Figure S4(f) for fitting residuals). From the fit, the same number of lifetimes are found on the skin as in solution, and the features captured in the EADS are relatively similar to those in solution. However, it is interesting to note that there are potentially favorable changes in relaxation lifetimes, presumably due to the interaction between the filter and the skin surface.

First, τ_1_ and τ_2_ for DPDT on the skin are shorter than that seen in solution: 1.09 ± 0.10 and 55.4 ± 1.6 ps, respectively. These lifetimes were assigned in solution to vibrational and geometrical relaxation to a local minimum (τ_1_) before further vibrational and geometrical relaxation to a potential energy minimum (τ_2_) from which fluorescence takes place. Due to the similarities in the EADS seen compared to those in solution, they are assigned to similar processes on the skin. It is somewhat surprising, however, that the lifetimes for these processes are shorter for samples on the skin when compared to those in solution since, for the skin surface environment, one would expect the motion to be restricted, and hence, vibrational relaxation would be slower. Nevertheless, our experimental observations indicate the contrary. It is plausible that the more restrictive surface environment might result in any minima being closer to the Franck–Condon region, requiring less motion and hence taking less time for the excited-state population to reach them. This is potentially supported by the reduced spectral shifts as the molecule relaxes (as seen on consecutive EADS) on the skin compared to that in solution, possibly indicating fewer structural changes are required to reach the minima. Alternatively, if, in solution, the considerably large solvation shell plays a significant role in τ_2_, a lack of solvent shell here could result in faster relaxation to the energy minimum from where fluorescence takes place.

The final lifetime for DPDT, τ_3_, on the skin is nearly half of that seen in solution at 616.1 ± 6.5 ps and is assigned, analogously to τ_3_ in solution, as fluorescence. A variety of explanations can rationalize this reduced fluorescence lifetime. First, an enhanced Franck–Condon overlap from the populated electronic excited state to the ground electronic state, as a result of the interaction with the skin surface, would increase the probability of fluorescence and, in turn, reduce its lifetime. Interaction with the skin surface could also open a nonradiative decay pathway within DPDT, which competes with radiative decay, effectively becoming the rate-limiting step and thus reducing the observed fluorescence lifetime. Alternatively, the skin itself may act as a quencher, with energy transferring from DPDT to the skin, opening an alternative decay pathway that competes with fluorescence, a process that would be highly detrimental in a real-world setting. These observations are also valid for PBSA on the skin, for which we propose a relaxation mechanism similar to that in solution despite fewer spectral shifts in ESA and SE as the molecule relaxes, with a nearly 2-fold reduction in fluorescence lifetime (Table 1 and SI Figure S5).

These results highlight how environmental conditions can potentially alter UV filter photodynamics in a favorable way. Overall, DPDT and PBSA exhibit potentially beneficial photodynamical changes as UV filters when applied to the skin for sunscreen formulations. The red-shifted absorbance improves UVA coverage, and more importantly, a reduced excited-state lifetime decreases the probability of undesired processes that could lead to the generation of radicals and other reactive species that are potentially detrimental. Nevertheless, PBSA and DPDT still undergo radiative relaxation and remain in the excited state for a prolonged period of time, both of which pose a potential risk to human health when applied to the skin, as previously discussed.

### Troxerutin Fluorescence Quenching

Given that, as outlined previously, fluorescence is a nonideal relaxation pathway for UV filters intended for use in sunscreen formulations, fluorescence quenchers are often needed to facilitate faster-excited-state relaxation in these filters. One such fluorescence quencher is troxerutin (see SI Figure S2 for structure). Symrise has patented the use of troxerutin for the quenching of DPDT emission;^29^ in this study, the quenching effects of troxerutin are investigated for both PBSA and DPDT. In water, troxerutin has a broad absorption with a λ_max_ of 348 nm but a relatively low ε of ∼16,500 M^–1^ cm^–1^ (Figure 2). TEAS measurements for troxerutin, equivalent to those just presented for PBSA and DPDT, reveal two lifetimes when excited at the λ_max_ of PBSA and DPDT in solution (SI Figure S6). One that is relatively short (<10 ps), which we attribute to ground-state recovery, and a feature that extends beyond our experimental time window of 1.8 ns resembles some possible long-lived excited-state population or photoproduct formation. Furthermore, emission studies reveal low emission for troxerutin, proving that troxerutin itself does not dissipate absorbed energy radiatively (SI Figure S7).

Initially, the addition of troxerutin proved promising for quenching the emission of both PBSA and DPDT in solution, demonstrating a successive reduction in emission intensity being dependent on troxerutin concentration (see SI Figure S7). However, when accounting for the primary and secondary inner filter effect (absorption of the excitation and emission), troxerutin surprisingly does not appear to show any significant fluorescence quenching in solution (see SI Figure S8), with any successive reduction in emission intensity observed resulting from the inner filter effect and not quenching.^42^ This inner filter effect can be visually observed for DPDT with troxerutin for concentrations similar to that used in commercial sunscreen formulations (SI Figure S9). Further TEAS, fluorescence lifetime, and absorption measurements support this finding, showing no evidence for a change in photodynamics or interaction between troxerutin and PBSA or DPDT (SI Figures S10–S12).

Overall, these solution-phase experiments indicate that troxerutin does not significantly quench the fluorescence of either PBSA or DPDT in water (at least at concentrations of 500 μM or lower). It is unknown why this occurs, but it could be that the complexation equilibrium constant is too low for a static quenching mechanism or that the distribution of donor–acceptor distances is distorted away from that required for a mechanism such as Förster resonance energy transfer (FRET). Both potentially result from a large solvent shell on the negatively charged PBSA and DPDT. It is possible that, at very high concentrations, FRET or a quenching mechanism such as Dexter energy transfer (DET), which requires very short donor–acceptor distances, could still occur.^43^ However, at those concentrations, inner filter effects would make it impossible to observe any such process with these experiments.

On the skin, in a more realistic setting, the absorption profile of troxerutin, in comparison to solution, has a broad red-shifted absorption with a λ_max_ of 357 nm (see SI Figure S13). Furthermore, it exhibits relaxation dynamics similar to that in solution with an excitation wavelength of 346 nm (λ_max_ of DPDT) but with a very low signal strength (SI Figure S14). TEAS of troxerutin on the skin at the λ_max_ of PBSA was not carried out due to the already very low signal strength at 346 nm (assumed to be similar, as seen in solution, but with a lower signal intensity).

**Figure 7:**
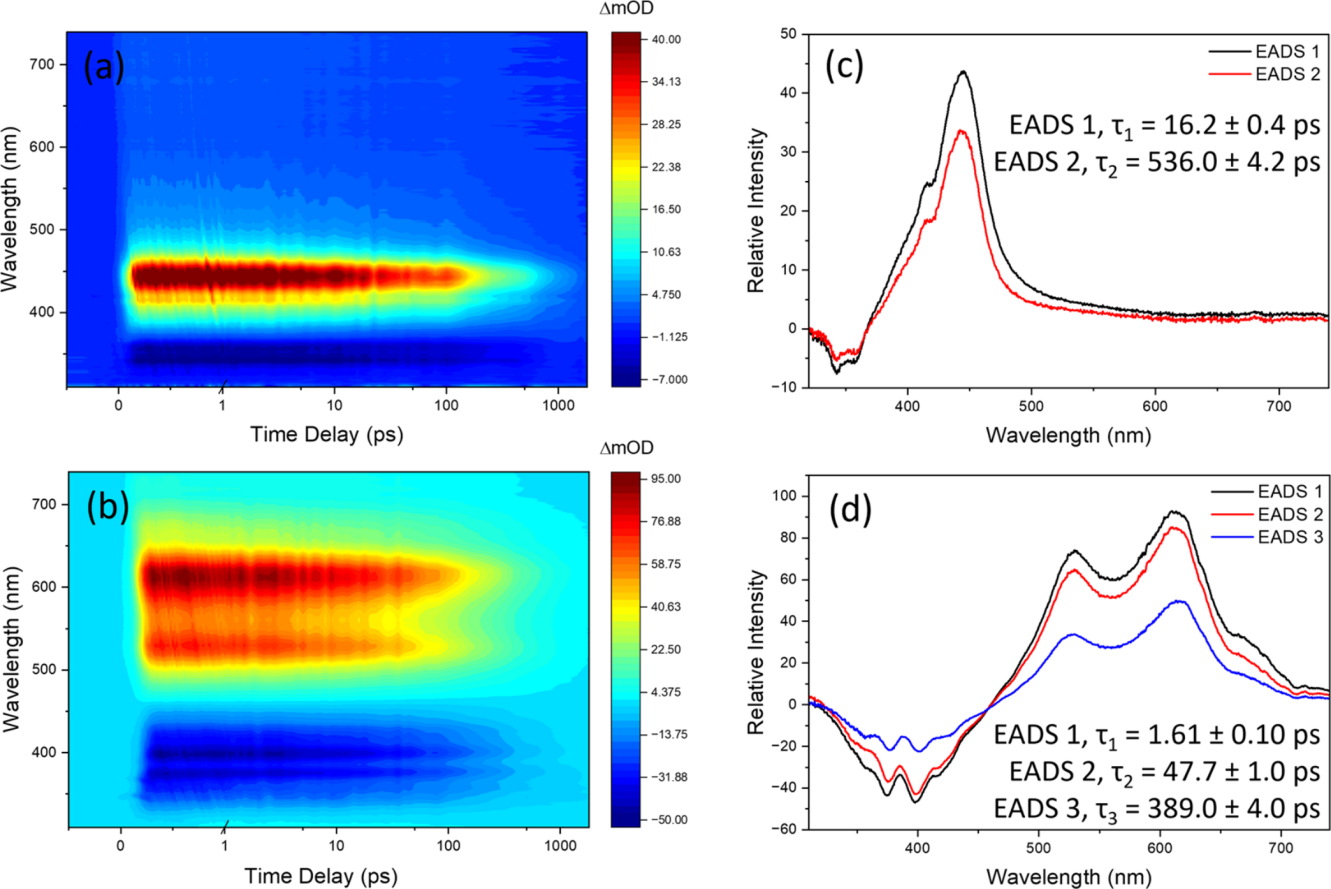
TEA spectra presented as false color heatmaps for (a) PBSA and (b) DPDT on human skin mimic with troxerutin, following excitation at their respective λ_max_. There is a 1:1 ratio of PBSA to troxerutin and a 1:3 ratio of DPDT to troxerutin. In both cases, the time delay is linear up to 1 ps and logarithmic from 1 to 1800 ps. The EADS obtained from the global sequential fitting model in the Glotaran software package are shown in parts (c, d) for PBSA and DPDT, respectively. The corresponding lifetimes for EADS are presented for each graph.

Due to the inconsistent local sample concentrations and random amounts of scatter, it was unreliable to compare different ratios of fluorophore to quencher to investigate the extent of quenching. As such, we focus on whether quenching is observed and how it is presented. Figure 7a,b presents the TEA spectra of PBSA and DPDT with the addition of troxerutin on the skin, respectively. The concentration ratio of PBSA to troxerutin was 1:1, while for DPDT to troxerutin, the ratio was changed to 1:3 to observe a reliable and conclusive observable change in dynamics. The EADS and corresponding lifetimes obtained from the global sequential fitting model are shown in Figure 7c,d for PBSA and DPDT, respectively (see SI Figure S15 for fitting residuals). Overall, the spectra show features and dynamics largely comparable to that seen for PBSA and DPDT on the skin without the addition of troxerutin (Figure 6 and SI Figure S5). There are, however, significant differences in the lifetimes, in contrast to what was observed in solution with the addition of troxerutin, further demonstrating the importance of environmental conditions.

Starting with the lifetimes attributed to nonradiative relaxation, τ_1_ for PBSA shows a significant reduction in lifetime from ∼45 ps without the addition of troxerutin to 16.2 ± 0.4 ps with the addition of troxerutin on the skin. This stark difference suggests a substantial interaction between troxerutin and PBSA, resulting in a significantly altered potential energy landscape for PBSA, which now facilitates a faster relaxation to the minimum from which fluorescence takes place. However, these differences in photodynamics could also simply be a result of troxerutin’s own dynamics, which includes a fast component (∼10 ps, see SI Figure S14), influencing the fitting (the spectra of troxerutin were not subtracted). For DPDT, τ_1_ and τ_2_ appear largely unchanged to those seen without the addition of troxerutin (Figure 6 and Table 1), and any minor differences likely result from the influence of troxerutin’s dynamics.

The most interesting changes observed in the TEAS of these UV filters on the skin, in the presence and absence of troxerutin, relate to the fluorescence lifetimes: τ_2_ = 536.0 ± 4.2 ps for PBSA and τ_3_ = 389.0 ± 4.0 ps for DPDT. In each case, the fluorescence lifetimes in the presence of troxerutin are nearly half those observed in its absence (Table 1), in stark contrast to the lack of lifetime change seen with the addition of troxerutin in the solution.

From the change in fluorescence lifetimes, it is clear that fluorescence quenching occurs on the skin, even if we do not see evidence for the same process taking place in solution. What is yet unknown are the mechanism of quenching and why it is unique to the skin. Collisional quenching, a type of dynamical quenching, can be ruled out on the surface of the skin due to the lack of mobility for diffusion.^43^ Furthermore, by definition, static quenching does not occur due to the observed change in lifetime upon the addition of the quencher. This leaves two main possible quenching mechanisms: FRET and DET.

DET requires orbital overlap, which decreases exponentially with donor–acceptor distance, requiring extremely close distances of ∼0.5–1 nm to occur.^44^ On the other hand, FRET occurs through space via long-range dipole–dipole coupling at distances of ∼1–10 nm.^44^ Since the solutions applied to the skin mimic are left to dry, the concentration on the skin surface will be considerably high, likely containing the distances required for both DET and FRET to occur. Furthermore, both DET and FRET require overlap between the donor’s emission spectrum and the acceptor’s absorption spectrum, a requirement which is met when pairing either PBSA or DPDT with troxerutin (see SI Figure S13). In fact, there is a much greater overlap between PBSA’s emission and troxerutin’s absorption, which might explain why only a lower ratio of 1:1 (PBSA to troxerutin, cf. 1:3 DPDT to troxerutin) was required to observe a reliable and conclusive observable change in dynamics.

From these deductions, we propose that troxerutin quenches the fluorescence of PBSA and DPDT on the surface of the skin predominantly via a DET or FRET quenching mechanism. Both mechanisms ultimately provide an additional nonradiative decay pathway for the trapped population in the excited state of PBSA and DPDT, thus reducing the fluorescence lifetime. Unfortunately, due to the surface concentration and surface concentration ratios between the fluorophore and quencher being unreliable, an accurate comparison between different fluorophore and quencher ratios is not possible. As such, it is unknown how efficient troxerutin is at quenching these molecules, but we can likely infer that quenching is more efficient for PBSA than for DPDT due to the greater spectral overlap. As to why quenching is observed on the surface of the skin and not in solution could result from the close proximity (and restricted motion) of molecules on the skin. In solution, the potentially large solvent shell reduces the possibility of the donor and acceptor getting within the required distances for these mechanisms to occur. Furthermore, particularly for FRET, there is an orientational dependence that is randomized in solution but not on the skin surface, where there could be an adsorption orientation relative to the surface that facilitates FRET.

## Conclusions

Following excitation to their λ_max_ in water, 2-phenylbenzimidazole-5-sulfonic acid (PBSA) and disodium phenyl dibenzimidazole tetrasulfonate (DPDT) undergo minor vibrational and geometrical relaxation before predominantly dissipating the remaining energy from the initially populated electronic excited state via fluorescence. The lifetime of fluorescence for PBSA and DPDT in water was found to be 1.68 and 1.14 ns, respectively, and both molecules exhibit large fluorescence quantum yields of 72 and 92%, respectively. Although the photodynamics of PBSA and DPDT are quite slow, which is undesirable in a commercial sunscreen formulation, both exhibit remarkable photostability with a <3% reduction in ultraviolet (UV) absorbance following 2 h of solar irradiation.

When applied to synthetic skin, PBSA and DPDT red shift in absorption and emission and both exhibit photodynamics similar to that seen in solution. The transient electronic absorption spectroscopy (TEAS) measurements presented here imply no overall change in the relaxation mechanism when applied to the skin, although a noticeable reduction in fluorescence lifetime is observed, which implies an improved Franck–Condon overlap between the excited state and the ground electronic state or that a possible nonradiative coordinate opened up due to interaction with the skin surface.

Regardless, the fluorescence of a sunscreen molecule in the UV region is detrimental to human health, and in the case of DPDT, the fluorescence of visible light has some negative cosmetic impacts by adding an artificial shine to the skin. As such, the commercially employed fluorescence quencher for DPDT, troxerutin, was evaluated for its ability to quench both PBSA and DPDT. In water, no significant evidence was found for fluorescence quenching. In stark contrast, troxerutin prompted a significant reduction in fluorescence lifetimes for PBSA and DPDT on the skin, and we conclude that a Dexter energy transfer (DET) or Förster resonance energy transfer (FRET) mechanism is responsible for the observed quenching.

Overall, this multipronged approach has demonstrated, for the first time, the overall relaxation dynamics for PBSA and DPDT in solution and on a skin mimic and revealed a DET or FRET fluorescence quenching mechanism for troxerutin with PBSA and DPDT on the skin mimic. We hope that from the findings revealed in this work, one can develop improved emission quenchers for UV filters. One approach for the development of emission quenchers is to reduce the average distance between the fluorophore and quencher by either chemically bonding them together, encapsulating them together in transparent nanoparticles, or utilizing a Coulombic attraction between the negatively charged fluorophore (such as PBSA and DPDT) and a cationic quencher. In the case of PBSA and DPDT, since both molecules require neutralizing to become soluble in water, a cationic quencher would serve a dual purpose of both quenching and solubilizing the UV filters.

## Experimental Section

PBSA, DPDT, and troxerutin were provided by Symrise (Holzminden, Germany). All solutions were made to a desired concentration (outlined below for the different experiments) in deionized water, and solutions containing PBSA or DPDT were then neutralized with NaOH to generate the corresponding salt. The synthetic skin samples were made by submerging a ∼2 cm^2^ square of a synthetic skin mimic, VITRO–CORNEUM (IMS Inc.), into a 2 mM solution of PBSA or DPDT with 0, 2, or 6 mM troxerutin (depending on the experiment) and subsequently removing excess residue using lens tissue before allowing it to dry (increasing the effective concentration on the surface to that of the solutions used). The resulting sunscreen skin samples were tested so that only those with an absorbance of ∼0.5 were taken forward for measurements.

The absorption spectra of PBSA, DPDT, and troxerutin in water at 20 μM and on the skin were taken using a Cary 60 spectrometer (Agilent Technologies). A 1 cm path-length quartz cuvette was used for the solutions, and the skin was placed on a 1″ diameter CaF_2_ window and housed in an optics mount. The steady-state solar simulator irradiation measurements used an Oriel LCS-100 solar simulator (spectrum shown in SI Figure S1), and the sample was positioned such that the irradiance was equivalent to one Sun, a unit of power flux which corresponds to the irradiance on the surface of the Earth on a clear summer day, i.e., approximately 1000 W/m^2^.

The steady-state emission spectra of PBSA, DPDT, and troxerutin in water at 20 μM and on the skin mimic were taken using a Horiba FluoroLog-3 instrument with an excitation wavelength set at the absorption maximum (λ_max_) of PBSA or DPDT, as obtained from the absorption spectra. Emission was collected at 90° to the sample excitation geometry. A quartz cuvette with a 1 cm path length was used for the solutions, and skin samples were placed on a 1″ diameter CaF_2_ window and positioned just off 45° relative to the incident radiation (by approximately ∼10–20°) to avoid scattered light. Emission lifetime measurements of each of the solution samples at their peak emission wavelength (as obtained from the emission spectra) were carried out with an excitation pulse from a NanoLED with a central wavelength of 318 nm.

The emission quantum yield for PBSA and DPDT in water at 20 μM was measured using an Edinburgh Instruments FS5 spectrofluorometer with the SC-30 integrating sphere module. The excitation wavelengths were set to the λ_max_ of PBSA or DPDT, and a 1 cm path-length quartz cuvette was used.

The TEAS setup and procedure have been outlined previously, and only details specific to these experiments are presented here.^45−49^ The pump pulse excitation wavelength was set to the λ_max_ of PBSA or DPDT. The pump pulse energy was ∼500 nJ, and the beam spot size was ∼350 μm at the sample. The probe pulse was a white light continuum that spanned ∼315–740 nm. A pump–probe time delay, Δ*t*, was generated using a motorized delay stage for a maximum Δ*t* of 1.8 ns. Two different sample and beam path setups were used for TEAS measurements: for samples in solution, a “vertical” setup was employed, whereas skin samples were studied in a “horizontal” arrangement. In the vertical setup, the solutions were delivered through a flow cell (Demountable Liquid Cell, Harrick Scientific Products Inc., Pleasantville, NY) between two 25 mm CaF_2_ windows (thickness: 1 mm front, 2 mm back) separated by a 100 μm poly(tetrafluoroethylene) spacer, which defined optical path length of the sample. Then, 500 μM solutions were recirculated between the CaF_2_ windows from a 25 mL reservoir using a peristaltic pump (Masterflex, Gelsenkirchen, Germany), and the flow cell was continuously translated in the *XY* plane perpendicular to the pump–probe geometry to ensure that fresh sample is interrogated with each set of pump–probe laser pulses. In the horizontal setup, the skin samples were placed on a 25 mm CaF_2_ window and mounted horizontally to prevent the sample from moving or dislodging. The sample was translated continuously. In this horizontal arrangement, the pump and probe beams are directed vertically down into the sample via a set of periscopes and collected by a mirror that directs transmitted light toward the detector. All other aspects of the “vertical” and “horizontal” setups are the same in both cases.

To obtain dynamical information from the TEAS experiments, the transient absorption spectra were fitted with a sequential  global fitting model in the software package Glotaran.^38^ The lifetimes obtained are linked to evolution-associated difference spectra (EADS) that represent the evolving spectral features relating to each lifetime extracted, and the quality of the fit can be assessed through the fitting residuals. This model includes multiple sequential exponential decays convoluted with a Gaussian to model the instrument response (IR). Here, the IR was estimated from solvent (water) scans whereby a kinetic trace at a particular wavelength was fitted with a Gaussian function, as well as a Gaussian multiplied by a sine function (see SI Figure S16). The instrument response for the skin samples was inferred from the solvent scans for the respective solutions of PBSA and DPDT. To improve the esthetics of the transient electronic absorption heatmaps, the data was chirp-corrected using the software package KOALA, which utilizes a third-order polynomial to model the dispersion.^50^

## Supporting Information Available

Solar simulator spectrum; emission lifetime measurements; emission quantum yield measurements; emission spectra; additional TEAS data; TEAS fitting residuals; troxerutin’s structure; absorption spectra; inner filter effect corrected emission spectra, and instrument response measurements (PDF)

## Abbreviations

UV: ultraviolet
PBSA: 2-phenylbenzimidazole-5-sulfonic acid
DPDT: disodium phenyl dibenzimidazole tetrasulfonate
TEAS: transient electronic absorption spectroscopy
λ_max_: absorption maximum
ε: molar extinction coefficient
AUCI: area under the curve indexes
TEA: transient electronic absorption
GSB: ground-state bleach
SE: stimulated emission
ESA: excited-state absorption
EADS: evolution-associated difference spectra
FRET: Förster resonance energy transfer
DET: Dexter energy transfer
IR: instrument response
ΔmOD: difference in milli optical density
