# Supplementary material for: Evaluating the Fluorescence Quenching of Troxerutin for Commercial UV Sunscreen Filters
Source: ACS Phys Chem Au. 2024 Oct 25;4(6):750–60. doi: 10.1021/acsphyschemau.4c00070 (PMC11613208; doi:10.1021/acsphyschemau.4c00070)
Supplement: Supplementary file 1 — pg4c00070_si_001.pdf [file pg4c00070_si_001.pdf]

Supporting Information for: Evaluating the Fluorescence Quenching of Troxerutin for Commercial UV Sunscreen Filters

Jack Dalton[1], Natércia d. N. Rodrigues[2], Daniel Berndt[3], Vasilios G. Stavros[4*]

[1]Department of Chemistry, University of Warwick, Gibbet Hill Road, Coventry, CV4 7AL, U.K.

[2]IBB-Institute for Bioengineering and Biosciences, Instituto Superior Técnico, Universidade de Lisboa, 1049-001 Lisboa, Portugal.

[3]Symrise AG, 37603 Holzminden, Germany.

[4]School of Chemistry, University of Birmingham, Birmingham, B15 2TT, U.K.

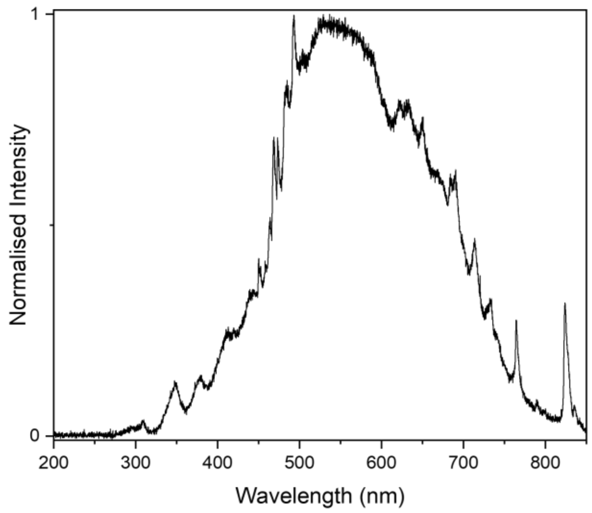

Figure S1. Solar simulator spectrum from Oriel LCS-100 used for irradiation measurements. In these irradiation measurements, the sample was positioned such that the irradiance was equivalent to one Sun, a unit of power flux which corresponds to the irradiance on the surface of the Earth on a clear summer day, *i.e.* approximately 1000 W/m^2.

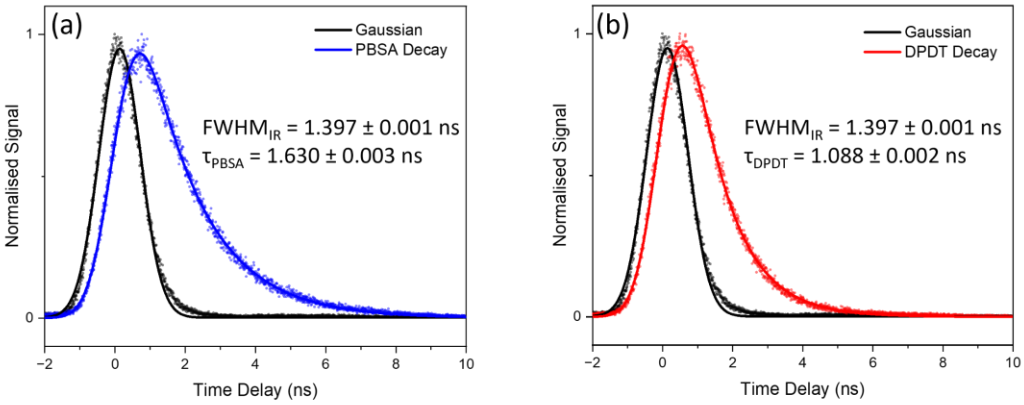

Figure S2. Emission lifetime measurements of PBSA (a) and DPDT (b) in water following nitrogen bubbling through the sample to remove oxygen. The excitation wavelength was 318 nm and the emission was detected at the emission maximum, 334 nm and 389 nm for PBSA and DPDT respectively (see Fig. 3 (a) in the main manuscript). The instrument response (IR) is shown by the black dots and was fitted using a Gaussian (black line) of which the full width half maximum is shown for each plot (FWHM$_{IR}$). The decay of PBSA (blue dots) and DPDT (red dots) was fit with a mono-exponential decay convoluted with a Gaussian. The errors shown relate to one standard error between the fit and the raw data. The returned lifetimes coincide with the decays obtained without nitrogen bubbling (Fig. 3 (b) in the main manuscript), which are 1.613 ns and 1.081 ns for PBSA and DPDT, respectively.

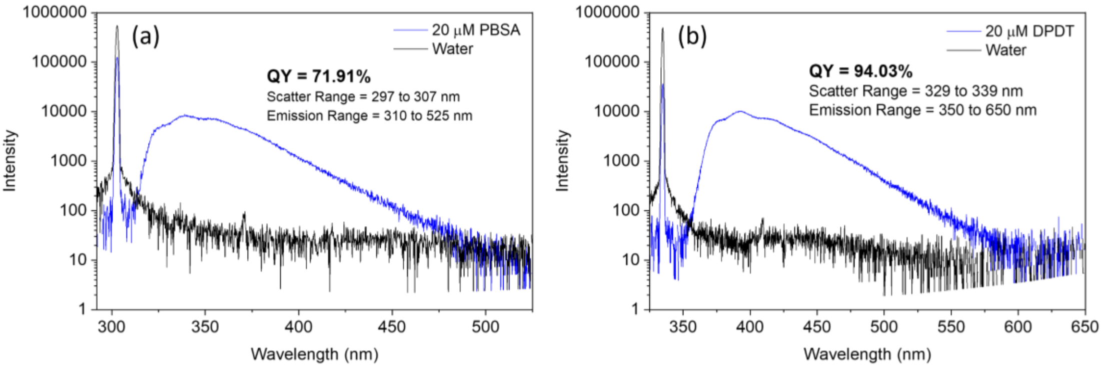

Figure S3. Pump scatter and emission (collected with an integrating sphere) of (a) PBSA and (b) DPDT in water at 20 μM following excitation at 302 and 334 nm respectively (blue). The corresponding pump scatter and emission of water is shown in black. The fluorescence quantum yield (QY) is shown for each graph in addition to the scatter and emission range used to calculate the QY.

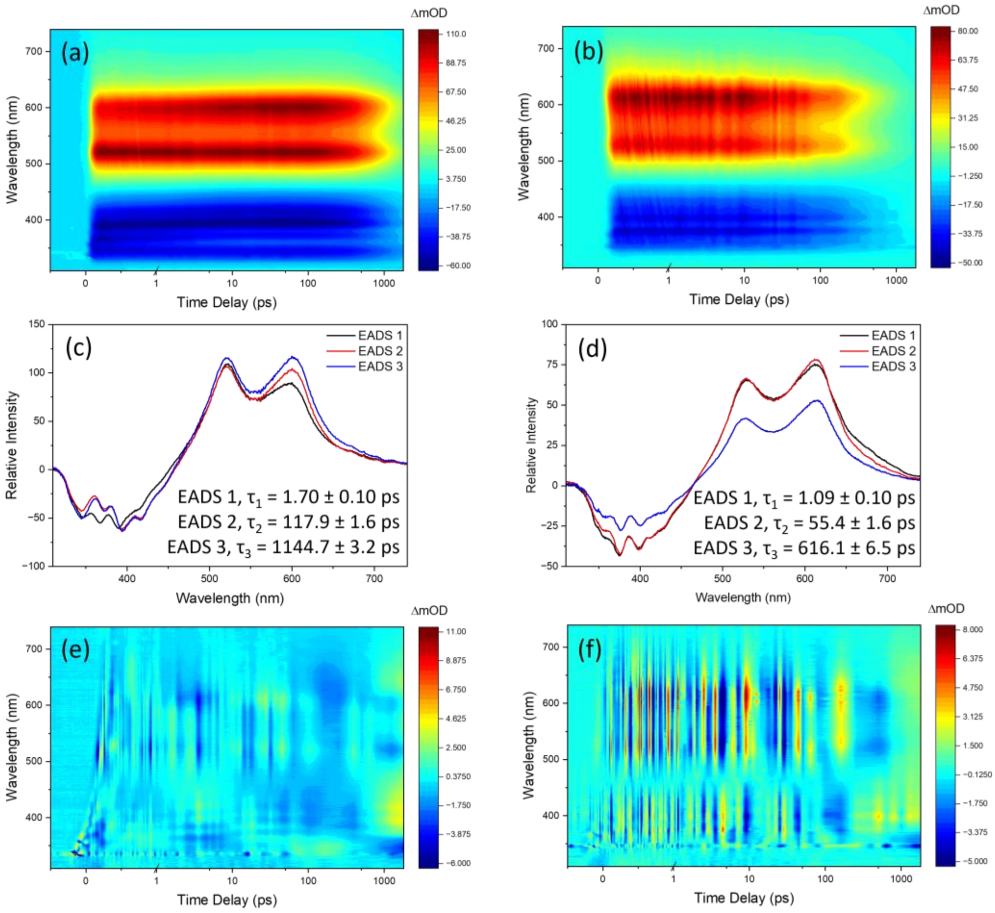

Figure S4. Transient electronic absorption (TEA) spectra presented as false colour heatmaps for DPDT (a) in water at 500 μM (neutralised with NaOH) and (b) on a skin mimic, following excitation at 334 and 346 nm respectively. The evolution associated difference spectra (EADS) obtained from the global sequential fitting model in the Glotaran software package are shown in (c) and (d) for DPDT in water and on skin, respectively. The corresponding lifetimes for the EADS are presented in (c) and (d). (e) and (f) present the fitting residuals for DPDT in water and on skin, respectively (the ΔmOD limits are set to 10% of the corresponding TEAS heatmaps to aid in assessing the quantity of the fit). For all heatmaps, the time delay is linear up to 1 ps and logarithmic from 1 to 1800 ps.

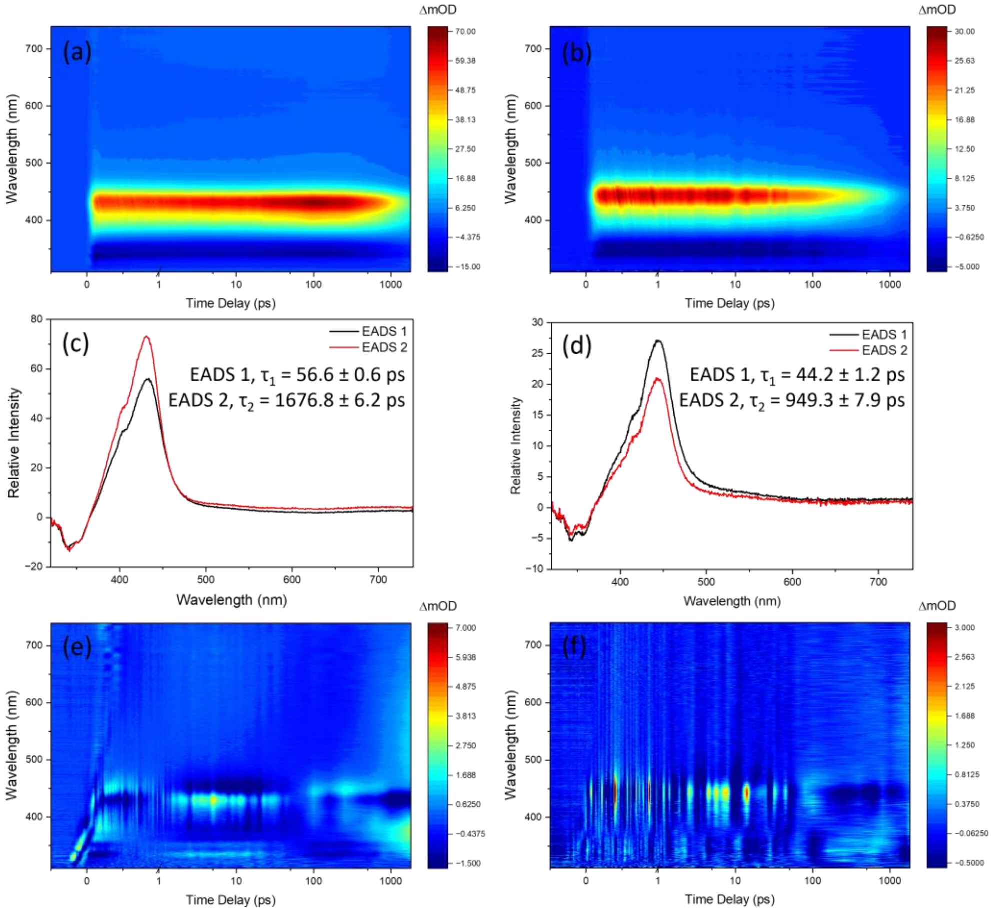

Figure S5. TEA spectra presented as false colour heatmaps for PBSA (a) in water at 500 µM (neutralised with NaOH) and (b) on a skin mimic, following excitation at 302 and 307 nm respectively. The EADS obtained from the global sequential fitting model in the Glotaran software package are shown in (c) and (d) for PBSA in water and on skin, respectively. The corresponding lifetimes for the EADS are presented in (c) and (d). (e) and (f) present the fitting residuals for PBSA in water and on skin, respectively (the ΔmOD limits are set to 10% of the corresponding TEAS heatmaps to aid in assessing the quantity of the fit). For all heatmaps, the time delay is linear up to 1 ps and logarithmic from 1 to 1800 ps.

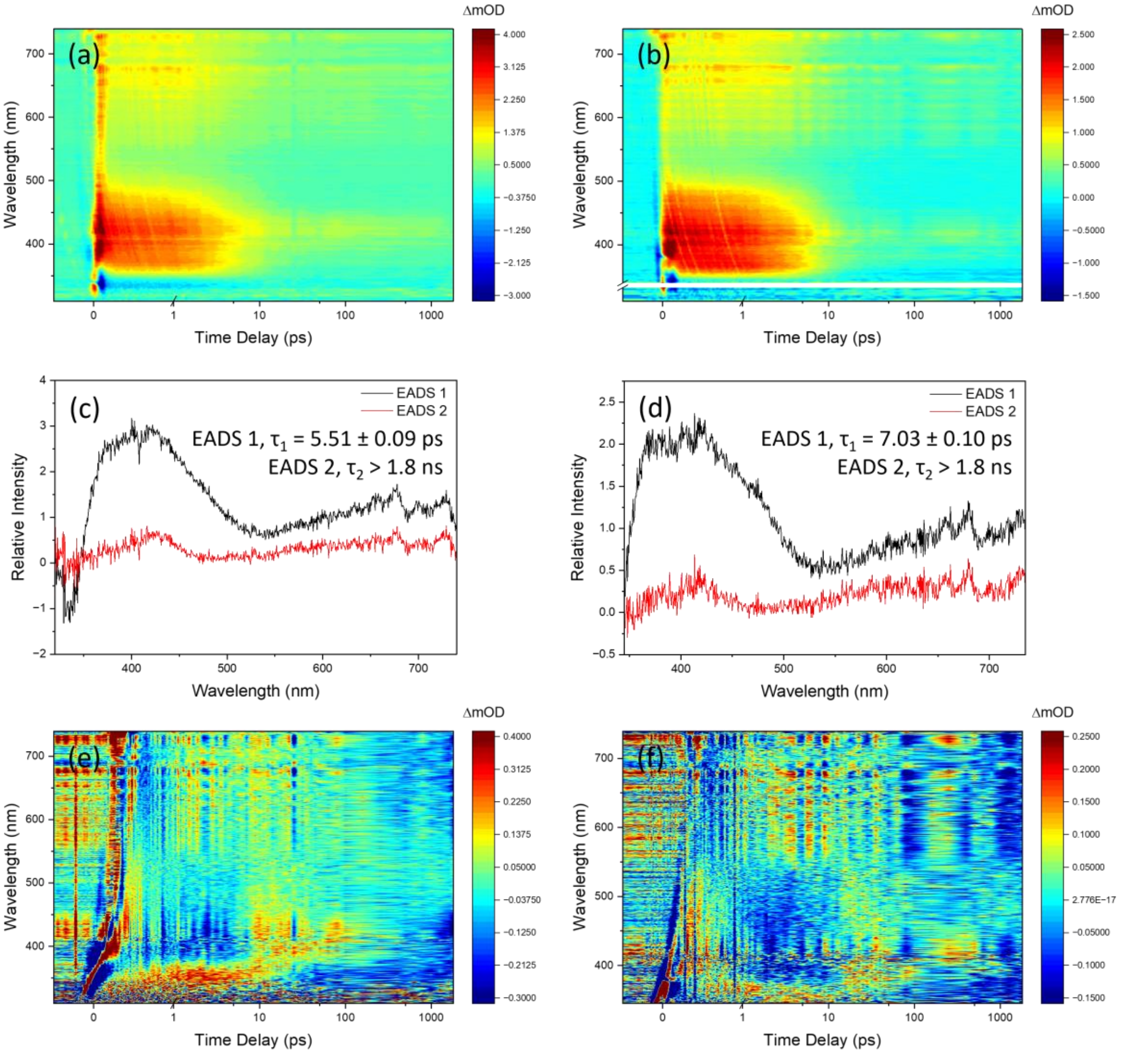

Figure S6. TEA spectra presented as a false colour heatmap for troxerutin at 500 µM in water following excitation at (a) 302 nm and (b) 334 nm. The EADS and corresponding lifetimes obtained from the global sequential fitting model in the Glotaran software package are shown for troxerutin photoexcited at (c) 302 nm and (d) 334 nm. (e) and (f) present the fitting residuals for troxerutin photoexcited at 302 nm and 334 nm, respectively (the ΔmOD limits are set to 10% of the corresponding TEAS heatmaps to aid in assessing the quantity of the fit). For the heatmaps, the time delay is linear up to 1 ps and logarithmic from 1 to 1800 ps.

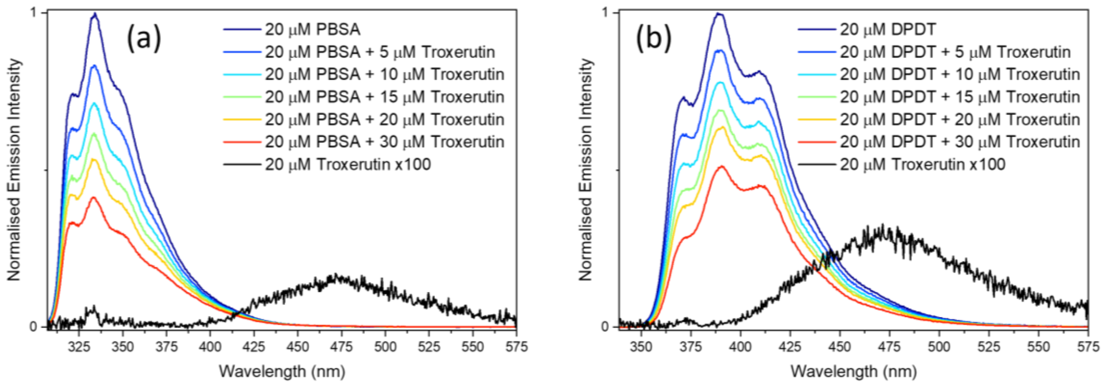

Figure S7. Emission spectra of (a) PBSA and (b) DPDT in water at 20 μM following excitation at their respective λ_{max} of 302 and 334 nm, with various concentrations of troxerutin added. Additionally shown in (a) and (b) is the emission spectrum of troxerutin at 20 μM following excitation at the λ_{max} of PBSA and DPDT, respectively (multiplied by 100 for visual aid).

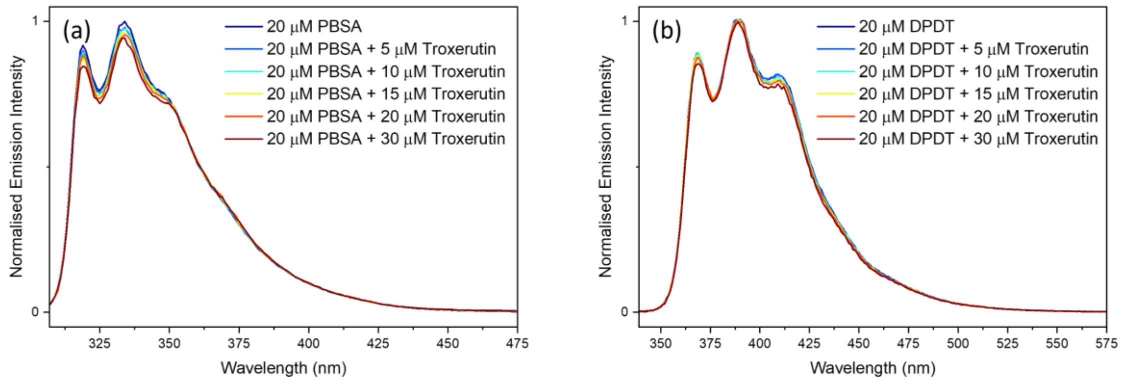

Figure S8. Corrected emission spectra of (a) PBSA and (b) DPDT at 20 μM following excitation at 302 and 334 nm respectively, with various concentrations of troxerutin added. The observed emission at each wavelength in SI Fig. S7 is multiplied by $10^{(A_{ex}+A_{em}(\lambda))/2}$; were, A_{ex} is the absorbance at the excitation wavelength to account for the primary inner filter effect and, $A_{em}(\lambda)$ is the absorbance at each emission wavelength to account for the secondary inner filter effect. As the concentration of troxerutin increases, A_{ex} increases, reducing the number of excited fluorophores and, $A_{em}(\lambda)$ increases, reducing the observed emission.

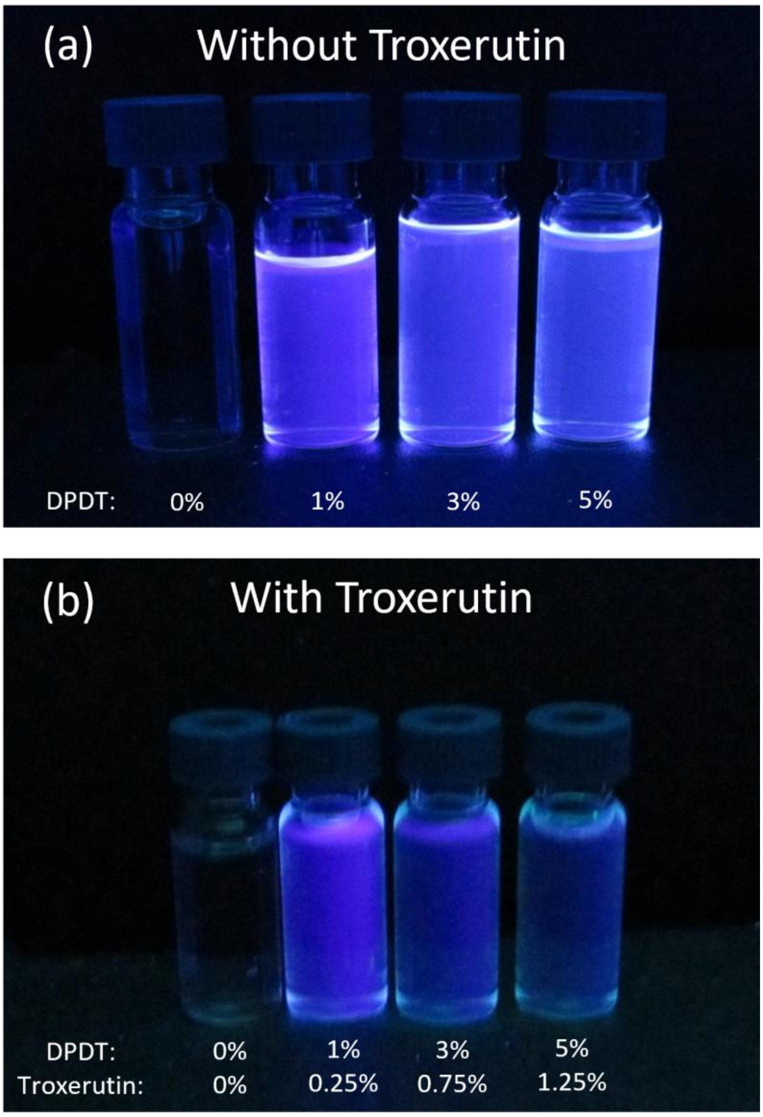

Figure S9. Qualitative study to visually demonstrate the inner filter effect for DPDT with troxerutin in solution at concentrations close to those used in commercial sunscreen formulations. (a) presents, from left to right, solutions of solvent (baseline comparison), 1% w/w DPDT, 3% w/w DPDT and 5% w/w DPDT. (b) presents in the same order from left to right, the DPDT solutions shown in (a) but with the addition of 0%, 0.25%, 0.75% and 1.25% w/w troxerutin. The solvent used is a pH 7 buffer:ethanol (80:20). As seen, with increasing concentrations there is a significant and noticeable reduction to the observed fluorescence due to the inner filter effect. To note, 5% w/w DPDT is over 2 orders of magnitude greater than the concentration (500 μM) used for the solution phase TEA measurements (SI Fig. S10).

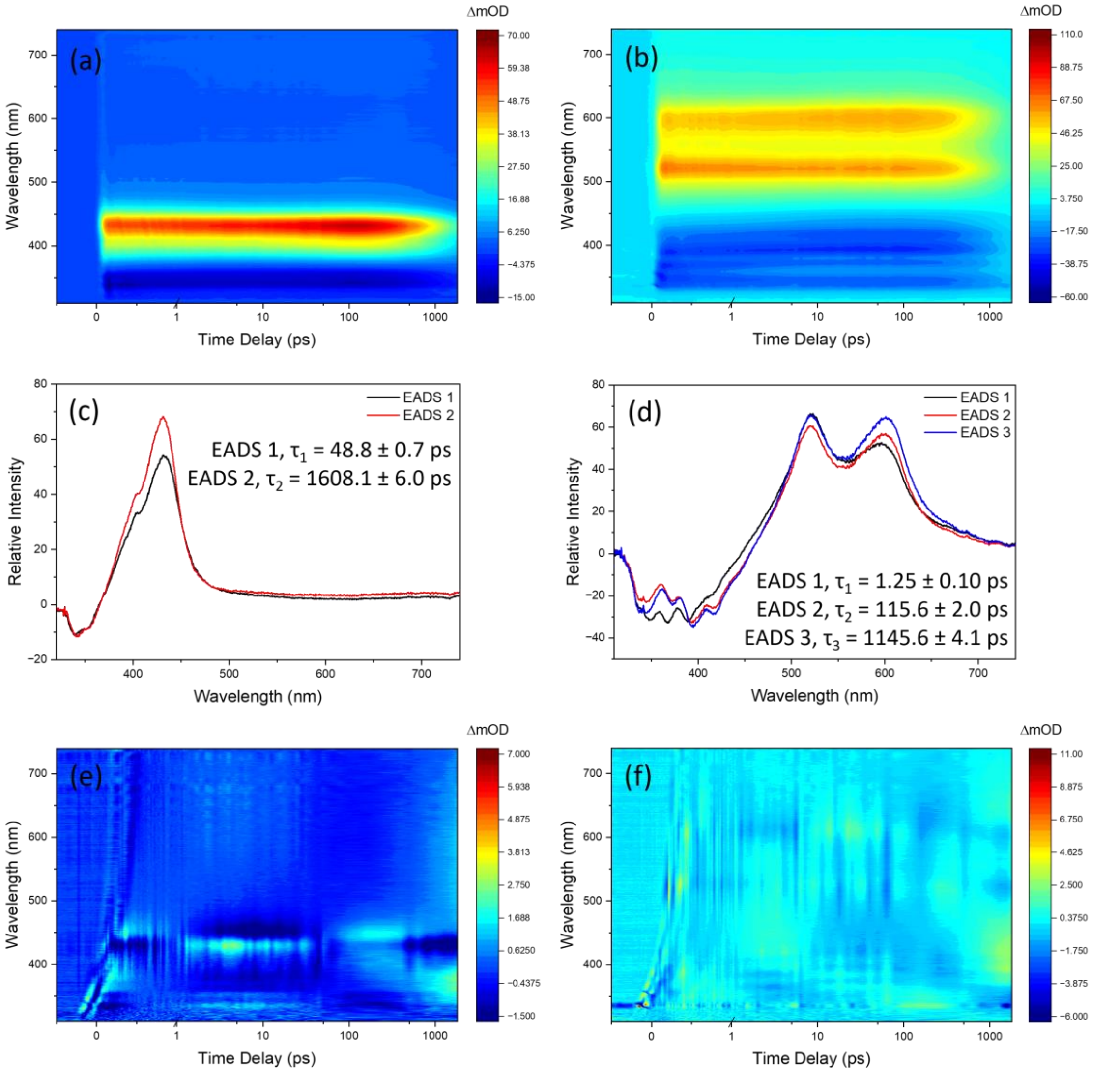

Figure S10. TEA spectra presented as false colour heatmaps for (a) PBSA and (b) DPDT at 500 µM in water (neutralised with NaOH) with the addition of 500 µM troxerutin and following excitation at 302 and 334 nm, respectively. To isolate the photodynamics of PBSA/troxerutin and DPDT/troxerutin from troxerutin alone, the 500 µM troxerutin spectra shown in SI Fig S6 were subtracted for the corresponding excitation wavelengths to produce (a) and (b). The spectra in SI Fig. S6 were carried out under identical experimental conditions. The EADS obtained from the global sequential fitting model in the Glotaran software package are shown in (c) and (d) for PBSA/troxerutin and DPDT/troxerutin, respectively. The corresponding lifetimes for the EADS are presented in (c) and (d). (e) and (f) present the fitting residuals for PBSA/troxerutin and DPDT/troxerutin, respectively (the ΔmOD limits are set to 10% of the corresponding TEAS heatmaps to aid in assessing the quantity of the fit). For all heatmaps, the time delay is linear up to 1 ps and logarithmic from 1 to 1800 ps. These results show a drop in signal for PBSA and DPDT but no change in the lifetimes and features observed. Thus, dynamic fluorescence quenching is not observed.

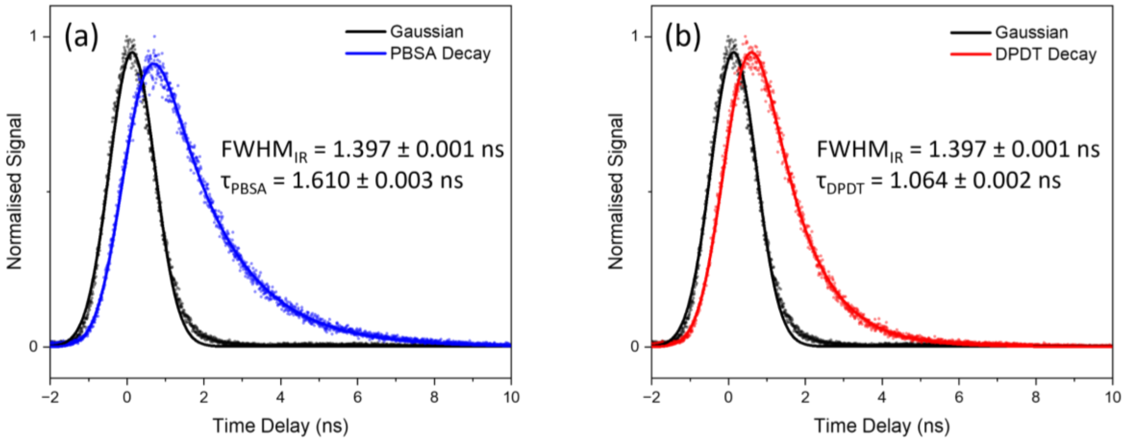

Figure S11. Emission lifetime at the peak emission of (a) PBSA and (b) DPDT at 20 µM with 20 µM troxerutin added following excitation at 318 nm. The instrument response is shown by the black dots and was fitted using a Gaussian (black line) of which, the full width half maximum is shown for each plot ($FWHM_{IR}$). The decay of PBSA (blue dots) and DPDT (red dots) was fit with an exponential decay convoluted with a Gaussian and the returned lifetimes are presented in each graph. The errors shown relate to one standard error between the fit and the raw data. These lifetimes coincide with the lifetimes found without the addition of troxerutin (Fig. 3 (b) in the main manuscript), which are 1.613 ns and 1.081 ns for PBSA and DPDT respectively. Thus, dynamic fluorescence quenching is not observed.

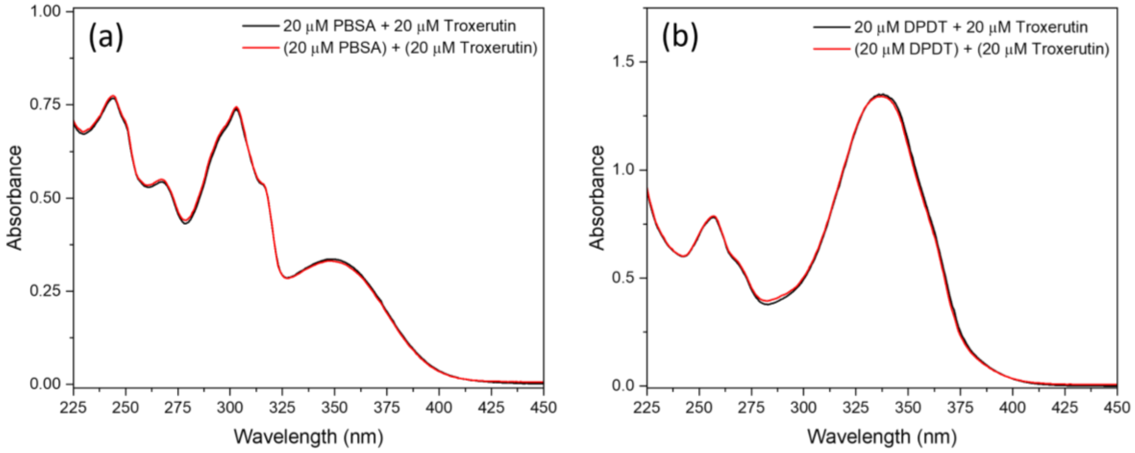

Figure S12. UV-Visible absorption spectra of (a) PBSA and (b) DPDT at 20 µM in water (neutralised with NaOH) with 20 µM troxerutin in the solution (black line) and, with the absorption spectrum of 20 µM troxerutin summed to the absorption spectrum of 20 µM PBSA or DPDT alone (red line). Both show no differences which indicates the lack of complexation between them. Thus, no ground state complex is formed for fluorescence quenching *via* a static quenching mechanism.

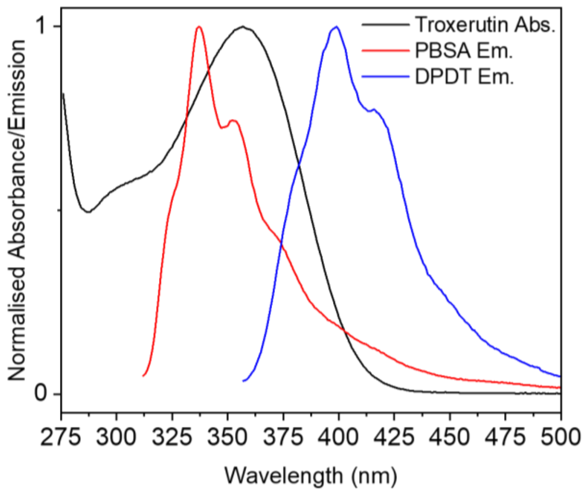

Figure S13. Normalised UV-visible absorption spectrum of troxerutin on a skin mimic, VITRO-CORNEUM® (black). The normalised emission spectra of PBSA (red) and DPDT (blue) on the skin mimic are also included to highlight the spectral overlap.

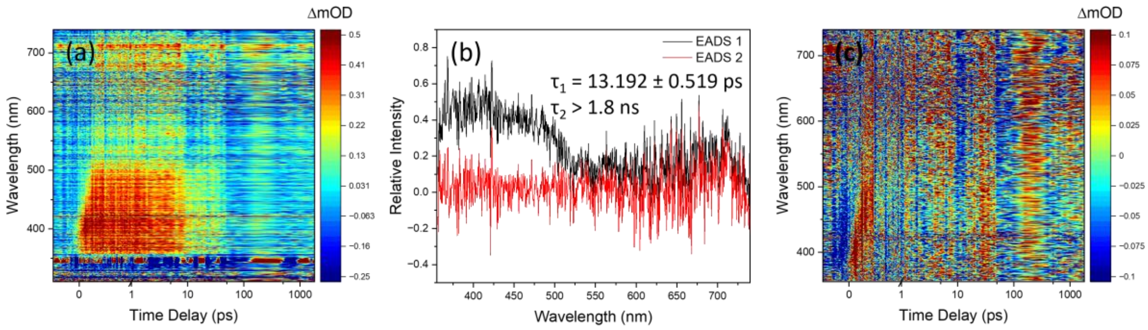

Figure S14. TEA spectra presented as false colour heatmaps for (a) troxerutin using a 8 mM solution on skin following excitation at DPDT's λ_{max} on skin, 346 nm. The EADS obtained from the global sequential fitting model in the Glotaran software package are shown in (b) along with the corresponding lifetimes. (c) presents the fitting residuals. For all heatmaps, the time delay is linear up to 1 ps and logarithmic from 1 to 1800 ps.

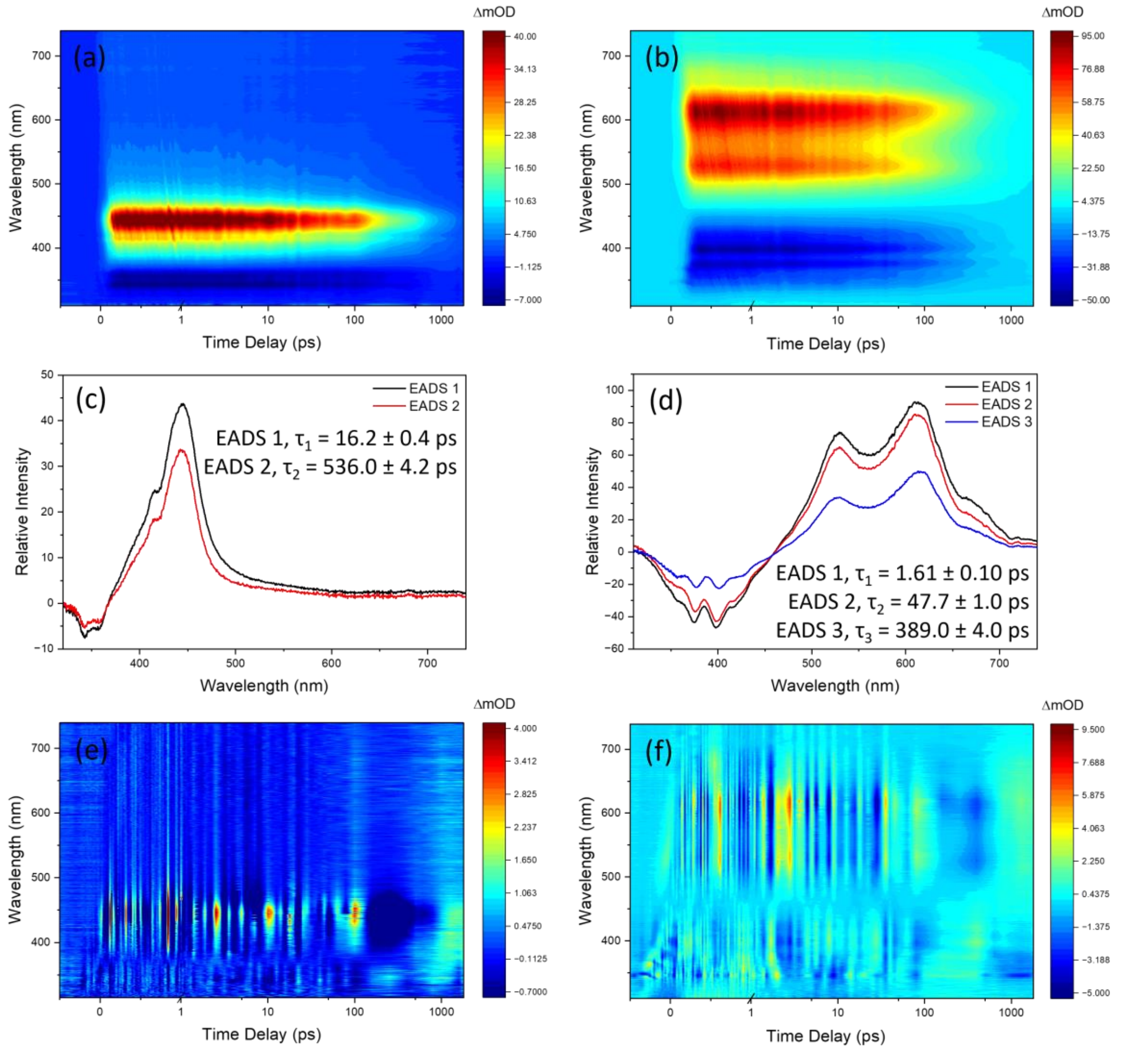

Figure S15. TEA spectra presented as false colour heatmaps for (a) PBSA and (b) DPDT on human skin mimic with troxerutin, following excitation at 307 nm and 346 nm, respectively. There is a 1:1 ratio of PBSA to troxerutin and a 1:3 ratio of DPDT to troxerutin. A 1:3 ratio with DPDT was chosen to observe a reliable and conclusive observable change in dynamics. The EADS obtained from the global sequential fitting model in the Glotaran software package are shown in (c) and (d) for PBSA and DPDT, respectively. The corresponding lifetimes for the EADS are presented for each graph. (e) and (f) present the fitting residuals for PBSA and DPDT, respectively (the ΔmOD limits are set to 10% of the corresponding TEAS heatmaps to aid in assessing the quantity of the fit). For all heatmaps, the time delay is linear up to 1 ps and logarithmic from 1 to 1800 ps.

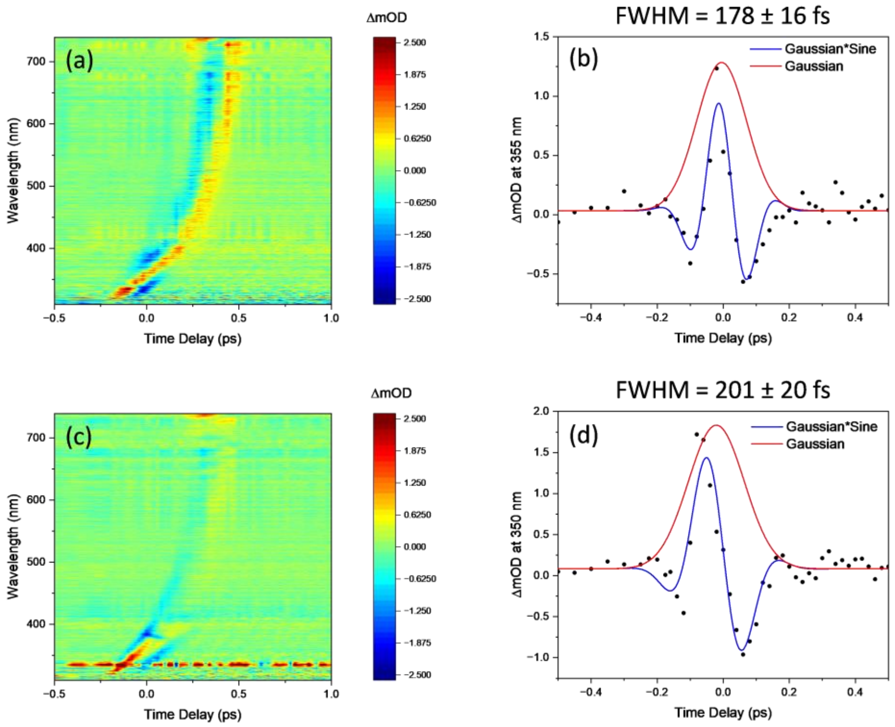

Figure S16. (a) TEA spectra false colour heatmap of water at a pump wavelength of 302 nm. (b) Transient slice at probe wavelength of 355 nm of (a). (c) Transient electronic absorption spectra false colour heatmap of water at a pump wavelength of 334 nm. (d) Transient slice at probe wavelength of 350 nm of (c). The blue line is a fit of a Gaussian multiplied by a sine function to capture the positive and negative features, and the full width half maximum (FWHM) of the Gaussian from this fit is shown above each plot along with the fitting error. These fluctuations are a result of nonlinear effects with the solvent.[1] The red line is a Gaussian with the FWHM determined from the blue line fit. This FWHM is an estimate for the instrument response (IR) of the transient electronic absorption spectroscopy measurements presented in this work and are determined to be approximately 178 ± 16 fs at a pump wavelength of 302 nm and 201 ± 20 fs at a pump wavelength of 334 nm.

References

(1) Lorenc, M.; Ziolek, M.; Naskrecki, R.; Karolczak, J.; Kubicki, J.; Maciejewski, A. Artifacts in Femtosecond Transient Absorption Spectroscopy. *Appl. Phys. B Lasers Opt.* **2002**, *74*, 19–27.
